# Methods for conducting a double-blind randomized controlled clinical trial of three days versus five days of amoxicillin dispersible tablets for chest indrawing childhood pneumonia among children two to 59 months of age in Lilongwe, Malawi: a study protocol

**DOI:** 10.1186/s12879-018-3379-z

**Published:** 2018-09-21

**Authors:** Amy Sarah Ginsburg, Susanne J. May, Evangelyn Nkwopara, Gwen Ambler, Eric D. McCollum, Tisungane Mvalo, Ajib Phiri, Norman Lufesi, Salim Sadruddin

**Affiliations:** 1 Save the Children, Seattle, Washington, USA; 2 University of Washington, Seattle, Washington, USA; 3 PATH, Seattle, Washington, USA; 4 Johns Hopkins School of Medicine, Baltimore, MD USA; 5 University of North Carolina Project Lilongwe Trust, Lilongwe, Malawi; 6 Malawi College of Medicine, Lilongwe, Malawi; 7 Malawi Ministry of Health, Lilongwe, Malawi; 8 World Health Organization, Geneva, Switzerland

**Keywords:** Childhood pneumonia, Chest indrawing, Amoxicillin, Treatment failure, Africa

## Abstract

**Background:**

Pneumonia is the leading infectious cause of death in children under 5 years of age around the globe. In addition to preventing pneumonia, there is a critical need to provide greater access to appropriate and effective treatment. Studies in Asia have evaluated the effectiveness of 3 days of oral amoxicillin for the treatment of fast-breathing pneumonia; however, further evidence is needed to determine if 3 days of oral amoxicillin is also effective for the treatment of chest indrawing pneumonia.

**Methods:**

This is a double-blind, randomized, non-inferiority trial with the objective to assess the effectiveness of shorter duration amoxicillin dispersible tablet (DT) treatment of chest indrawing childhood pneumonia in a malaria-endemic region of Malawi. The primary objective of this study is to determine whether 3 days of treatment with oral amoxicillin DT in HIV-uninfected Malawian children two to 59 months of age with chest indrawing pneumonia is as effective as 5 days of treatment. The study will enroll 2000 children presenting to Kamuzu Central or Bwaila District Hospitals in Lilongwe, Malawi. Each child will be randomized to either 3 days of amoxicillin DT followed by 2 days of placebo DT or 5 days of amoxicillin DT. Children in the study will be hospitalized for 48 h after enrollment and will have scheduled study visits at Days 2, 4, 6 and 14. Treatment failure by Day 6 is the primary outcome. We hypothesize that the rates of treatment failure will be similar in both arms and that 3 days of treatment will be non-inferior to 5 days of amoxicillin DT for chest indrawing pneumonia using a relative non-inferiority margin of 1.5. This trial was approved by the Western Institutional Review Board and Malawi College of Medicine Research and Ethics Committee.

**Discussion:**

Given the paucity of data from Africa, African-based research is necessary to establish appropriate duration of treatment with amoxicillin DT for chest indrawing childhood pneumonia in malaria-endemic settings in the region. An expanded evidence base will contribute to future iterations of World Health Organization Integrated Management of Childhood Illness guidelines.

**Trial registration:**

NCT02678195: Pre-results. Date registered February 9, 2016.

**Electronic supplementary material:**

The online version of this article (10.1186/s12879-018-3379-z) contains supplementary material, which is available to authorized users.

## Background

Pneumonia is the leading infectious cause of childhood mortality worldwide [1]. Estimates suggest that 920,000 children less than 5 years of age died from pneumonia in 2015, accounting for 15% of child deaths globally. In addition to preventing pneumonia, there is a critical need to provide greater access to appropriate and effective treatment. Studies in Asia have evaluated the effectiveness of 3 days of oral amoxicillin for the treatment of fast breathing pneumonia; [2–5] however, further evidence is needed to determine if 3 days of oral amoxicillin is also effective for the treatment of chest indrawing pneumonia. Given the paucity of data from Africa, African-based research is necessary to establish optimal treatment regimens for childhood pneumonia in the region. There is also a need for local studies in malaria-endemic settings in Africa using child-friendly amoxicillin dispersible tablets (DT), which the World Health Organization (WHO) has established as the optimal product formulation for first-line treatment of pneumonia in children less than 5 years of age [5]. The broad objective of this study is to provide scientific evidence assessing the optimal duration of treatment with amoxicillin DT for chest indrawing childhood pneumonia in a malaria-endemic setting in Malawi, Africa. We sought to determine whether 3 days of treatment with oral amoxicillin DT in HIV-uninfected children two to 59 months of age with chest indrawing pneumonia is as effective as 5 days of treatment. Our alternative hypothesis for this study is that 3 days is non-inferior to 5 days of amoxicillin DT treatment for chest indrawing pneumonia.

## Methods/design

### Study design and setting

**Fig. 1:**
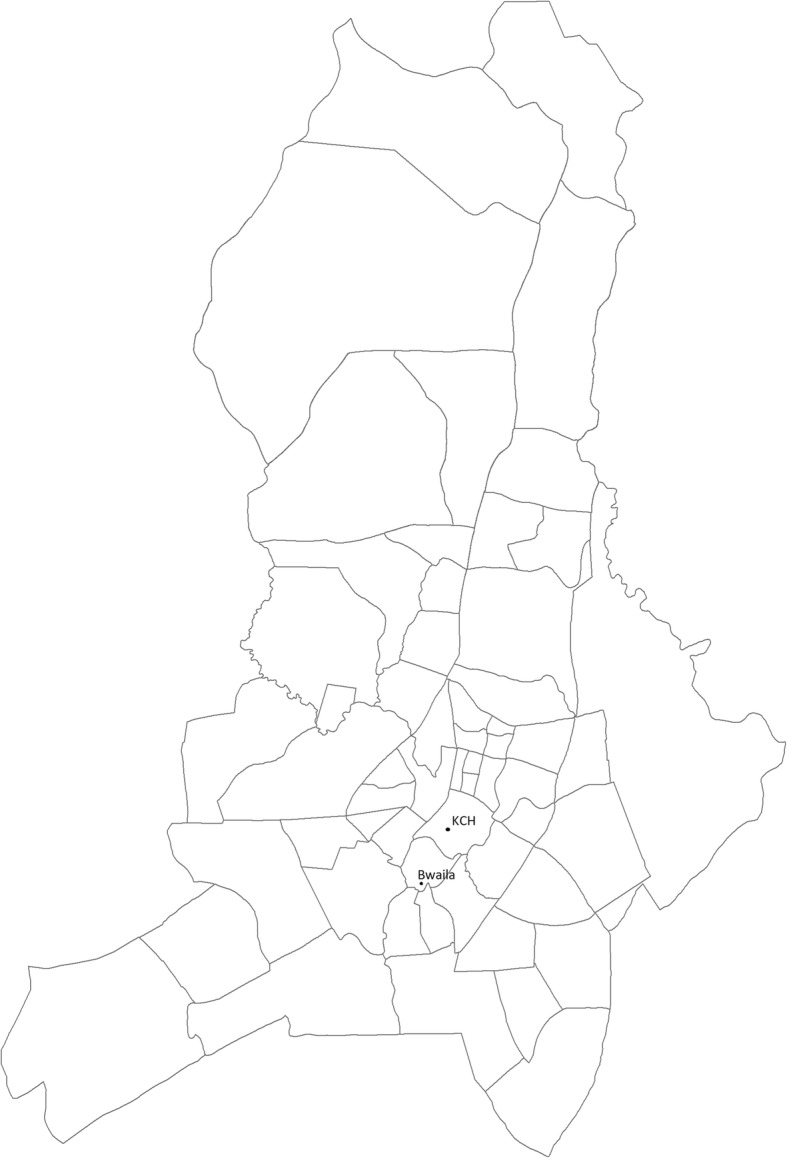
Map of study area in Lilongwe, Malawi

The primary objective of this prospective, double-blind, randomized controlled two-arm, non-inferiority trial is to determine whether or not treatment with 3 days of oral amoxicillin in HIV-uninfected children two to 59 months of age with chest indrawing pneumonia in a malaria-endemic region of Malawi is substantively less effective than 5 days of oral amoxicillin treatment. A non-inferiority design was chosen as there was a strong belief that placebo could not be expected to be more beneficial than amoxicillin with respect to the primary outcome of treatment failure by Day 6, but might be only slightly worse than amoxicillin. This study is conducted at Kamuzu Central Hospital (KCH) and Bwaila District Hospital (BDH) in Lilongwe, Malawi (Fig. 1). A 750-bed government facility, KCH is the primary referral hospital for the central region of Malawi, serving a population of approximately 5 million. BDH is the district hospital for Lilongwe with no inpatient facilities for children. Those children requiring inpatient care are referred to KCH.

### Study participants

**Fig. 2:**
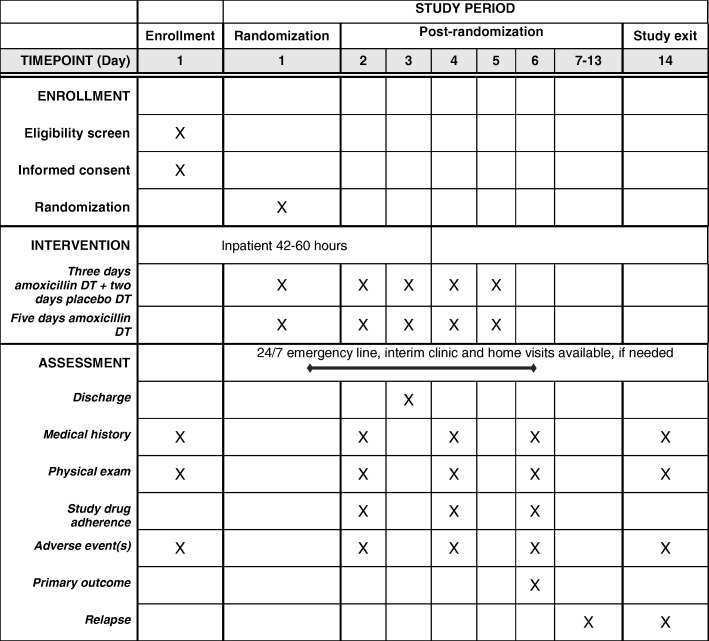
Schedule of enrollment, intervention, and assessment

Children aged two to 59 months with cough or difficulty breathing are recruited by trained hospital staff during routine intake and screening procedures in the hospitals’ outpatient departments, and referred to study staff for information on the study, written informed screening consent and additional screening to determine enrollment eligibility (Fig. 2). Screening procedures include assigning a participant identification number, collecting demographic and contact information and medical history, and assessing all eligibility criteria with a targeted physical exam, malaria rapid diagnostic testing, HIV rapid antibody testing, hemoglobin testing, and if wheezing, bronchodilator response testing.

Caregivers of those eligible children meeting the case definition of chest indrawing pneumonia (Table 1: eligibility criteria) are invited to participate by providing written informed consent (Additional file 1: Appendix 1) for enrollment. Final eligibility determination for enrollment depends on the results of the medical history, clinical examination, laboratory testing, appropriate understanding of the study, and completion of the enrollment consent process.

**Table 1:** Study definitions

Chest indrawing pneumonia	Cough less than 14 days or difficulty breathing AND visible indrawing of the chest wall with or without fast breathing for age
Fast breathing pneumonia	Cough less than 14 days or difficulty breathing AND fast breathing for age
Fast breathing for age	Respiratory rate > 50 breaths per minute (for children 2 to < 12 months of age) or > 40 breaths per minute (for children > 12 months of age)
Very fast breathing for age	> 70 breaths per minute (for children 2 to < 12 months of age) or > 60 breaths per minute (for children > 12 months of age).
Severe respiratory distress	Grunting, nasal flaring, and/or head nodding
Hypoxemia	Arterial oxyhemoglobin saturation (SpO_2_) < 90% in room air, as assessed non-invasively by a pulse oximeter
World Health Organization (WHO) Integrated Management of Childhood Illness (IMCI) general danger signs	Lethargy or unconsciousness, convulsions, vomiting everything, inability to drink or breastfeed
Severe acute malnutrition	Weight for height/length < − 3 SD, mid-upper arm circumference (MUAC) < 11·5 cm, or peripheral edema
Severe malaria	Positive malaria rapid diagnostic test (mRDT) with any WHO IMCI general danger sign, stiff neck, abnormal bleeding, clinical jaundice, or hemoglobinuria
HIV-1 exposure	Children < 24 months of age with a HIV-infected mother
Serious adverse event	Adverse event that: • Results in death • Is life threatening • Requires inpatient hospitalization or prolongation of existing hospitalization • Results in persistent or significant disability/incapacity • Is a medical event, based on appropriate medical judgment, that may jeopardize the health of the participating child or require medical or surgical intervention to prevent one of the outcomes listed
Eligibility criteria	
Inclusion criteria	• 2–59 months of age • Cough < 14 days or difficulty breathing • Visible indrawing of the chest wall with or without fast breathing for age • Ability and willingness of child’s caregiver to provide informed consent and to be available for follow-up for the planned duration of the study, including accepting a home visit if he/she fails to return for a scheduled study follow-up visit
Exclusion criteria	• Severe respiratory distress • Hypoxemia • Resolution of chest indrawing after bronchodilator challenge, if wheezing at screening examination • WHO IMCI general danger signs • Stridor when calm • HIV-1 seropositivity or HIV-1 exposure • Severe acute malnutrition • Possible tuberculosis (coughing for more than 14 days) • Hemoglobin < 8·0 g/dL • Severe malaria • Known allergy to penicillin or amoxicillin • Receipt of an antibiotic treatment in the 48 h prior to the study • Hospitalized within 14 days prior to the study • Living outside the study area • Any medical or psychosocial condition or circumstance that, in the opinion of the investigators, would interfere with the conduct of the study or for which study participation might jeopardize the child’s health • Any non-pneumonia acute medical illness which requires antibiotic treatment per local standard of care • Participation in a clinical study of another investigational product within 12 weeks prior to randomization or planning to begin participation during this study • Prior participation in the study during a previous pneumonia diagnosis
Treatment failure criteria	
Anytime on or before day 6	• Severe respiratory distress • Hypoxemia • WHO IMCI danger signs • Missing > 3 study drug doses due to vomiting • Change in antibiotics prescribed by a study clinician • Prolonged hospitalization or re-admission due to pneumonia • Death
At or after initial hospitalization discharge (between 42 and 60 h post-enrollment)	• Axillary temperature > 38 °C with chest indrawing
On day 6	• Axillary temperature > 38 °C • Chest indrawing

### Randomization and procedures

All randomization and study procedures are being conducted according to protocol version 8.0, March 14, 2016 (Additional file 2: Appendix 2). On Day 1, after study screening is complete and enrollment informed consent obtained, study staff perform the following procedures for enrollment: conduct physical exam; obtain vaccination history and collect additional socio-demographic information; assign randomization allocation; and administer initial study drug dose, study drug instructions, and concomitant medication prescriptions. Eligible children are immediately randomized by study staff following enrollment in a double-blinded manner in a 1:1 ratio using a priori computer-generated assignments to receive one of two treatments: 3 days of amoxicillin DT followed by 2 days of placebo DT (intervention) versus 5 days of amoxicillin DT (control). Amoxicillin DT is provided orally in 250 mg tablets and two divided doses based on age bands (500 mg/day for children 2 months up to 12 months, 1000 mg/day for children 12 months up to 3 years, and 1500 mg/day for children 3 years up to 5 years of age), the current WHO recommended therapy for HIV-uninfected children (Table 2) [5]. Study drug placebo DT and amoxicillin DT are identical in appearance, smell, taste, dispersion activity and packaging. Randomization is stratified by age groups using randomized blocks of size 2, 4 and 6. Separate randomization lists are generated by unblinded biostatisticians for the two hospitals (KCH and BDH), which are representative of two enrollment phases (initially, enrollment was done solely at KCH (phase 1) and then was transitioned to BDH (phase 2)). Unblinded study pharmacists receive the randomization lists and are responsible for assigning the treatment group and recording the blinded portion of the randomization code on case report forms and study drug packaging. With the exception of the unblinded study monitor, biostatisticians, and the data safety monitoring board (DSMB) members, all other study investigators, staff, and caregivers are blinded to each child’s assigned treatment group. The lists containing the link between the randomization code, treatment group, and participant identification number are maintained by the unblinded study pharmacists under lock and key and electronic encryption. Caregivers receive two study drug packs: a pack labeled with a red sticker containing study drug for Days 1 to 3, and a pack labeled with a blue sticker containing study drug for Days 4 and 5. Both packs are also labeled with the randomization code and study drug administration instructions. Blinded study staff administer the first study drug dose and demonstrate to the blinded caregiver with both verbal and pictogram instructions how to administer subsequent study drug doses appropriately in the hospital and at home. All subsequent doses administered during hospitalization are completed by the caregiver under careful observation by blinded study staff. If at any point during the trial an unanticipated need to unblind a child’s randomization allocation arises for reasons of child safety, the principal investigator, site investigators, study safety medical officer, and ethics committee will be notified and the instance will be documented.

**Table 2:** Study drug by age band

Age Band	Oral Amoxicillin/Placebo Dispersible Tablets (DT)	
	Number of 250 mg tablets, given two times daily	Total study drug administered per day
2 months up to 12 months	1	500 mg
12 months up to 3 years	2	1000 mg
3 years up to 5 years	3	1500 mg

**Fig. 3:**
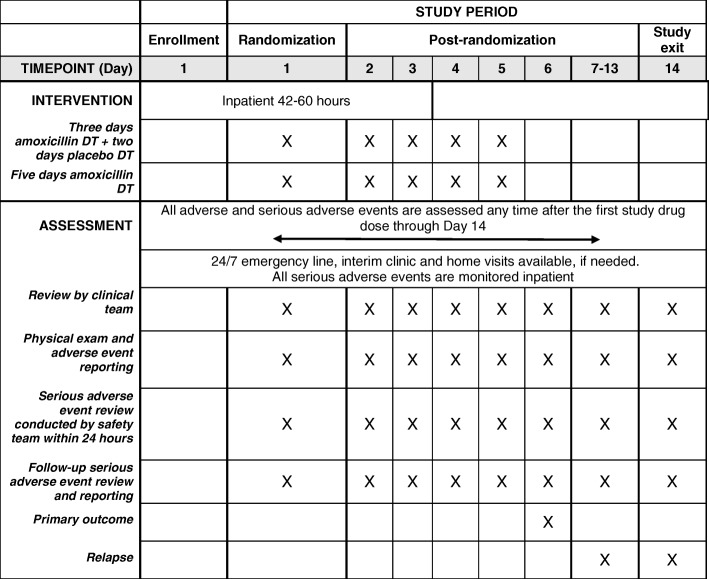
Framework to ensure child safety in the study

Recruitment, screening and enrollment occur at KCH or BDH, with BDH enrollees transferred to KCH for continued evaluation, observation and admission. Hospital observation or admission, and follow-up occur solely at KCH. To maximize study safety and allow continuous monitoring, all enrolled children are initially admitted to KCH for 2 days in a dedicated ward away from other sick children to minimize risk of nosocomial infection, and assessed for discharge on the morning of Day 3, 42 to 60 h after enrollment (Fig. 3). Additional diagnostic tests and medications including antibiotic treatment regimen changes for intercurrent illnesses are performed per KCH protocols. Discharge criteria are met if none of the criteria for treatment failure are present.

Caregivers are scheduled to bring their children for follow-up visits with the study staff on Days 2 (while under observation at KCH), 4, 6, and 14. Target dates for follow-up visits are calculated from Day 1, the date of randomization. All visits must occur on the calendar day on which they are initially scheduled or within 24 h afterwards, with the exception of the Day 14 visit, which can occur either 2 days before or after Day 14 and still be considered completed within the visit window. During follow-up visits, study staff review medical history and study drug adherence since the last study visit and perform a physical examination.

On hospital discharge and during follow-up study visits, caregivers are given detailed instructions by study staff on timing and administration of the study drug as well as signs and symptoms that should prompt an immediate call to study staff. A 24/7 study phone number is provided to the caregiver. In case of a no-show at scheduled follow-up visits, children are followed-up with home visits by study staff. During the 14 days of follow-up, all children are followed for treatment failure or relapse signs and symptoms by study staff who collect medical history and perform physical exams and monitor study drug adherence at all scheduled and unscheduled visits. Chest radiographs are obtained at the discretion of study clinicians and the local principal investigator makes the final clinical interpretation. Most children failing treatment during Days 1 to 6 or presenting with relapse during Days 7 to 14 are hospitalized and treated with second-line intravenous antibiotics. Once a second-line antibiotic is administered, the child is considered non-adherent to the randomized treatment.

For study drug adherence, completing 80% of all scheduled dose administrations is considered to meet treatment completion criteria (i.e., 8 out of 10 doses over the 5 days). If doses are missed due to non-adherence, no study action is taken beyond documenting the missed doses and counseling the caregiver on adherence and study drug administration. If a child vomits within 30 min of a dose, one repeat dose is attempted. If a child vomits within 30 min after three or more scheduled (i.e., not repeat) dose administrations, this is considered a treatment failure and that child is referred to care for a work-up of the vomiting cause and is prescribed a course of second-line antibiotics.

### Sample size

The sample size calculation is based on the primary endpoint. Because the treatment failure rate in the 5 day amoxicillin group is anticipated to be within the range of 4% to 12%, a relative non-inferiority margin is chosen as 1.5 times the failure rate in the 5 day amoxicillin group. The specific non-inferiority margin, 50% higher failure rate in the 3 day amoxicillin group compared to the 5 day amoxicillin group, was chosen after extensive discussions among the investigators and with external experts regarding what failure rate might be acceptable to clinicians for the 3 day amoxicillin group compared to the 5 day amoxicillin group, considering the anticipated potential failure rate in the 5 day amoxicillin group and potential for enrollment into the study. Adjusting for two formal interim analyses (with O’Brien-Fleming boundary for early non-inferiority [6] and Pocock boundary for early inferiority [7]) enrolling 2000 children (1000 per group) will provide 96.5% power if the failure rate is 12%, 84.2% power if the failure rate is 7%, and 64.8% power if the failure rate is 4% in the 5 day amoxicillin group with a failure rate in the 3 day amoxicillin group equal to that in the 5 day amoxicillin group. These power calculations take into account a drop-out rate of 5% and assume a one-sided alpha level of 0.025.

### Data collection and quality assurance

All study data is collected by clinical study staff using designated source documents or paper-based case report forms (Additional file 3: Appendix 3) which is then entered into electronic databases. Clinical research data is maintained through a combination of secure electronic data management system and physical files with restricted access to ensure confidentiality. Data related to study endpoints will be extracted from the electronic databases for statistical analyses. Three distinct study databases are maintained: the primary study database with study visit data, a safety database with serious adverse event assessments, and a database with participating children’s personally identifiable information. The database with identifiable information is maintained separately by the study site, while the designated contract research organization (CRO) maintains the primary study database and safety database. To ensure accuracy and completeness, data is routinely reviewed by the site quality control and assurance team as well as site monitoring visits by the co-investigators and CRO through quality assurance reviews, audits, and evaluation of the study safety and progress. Standard Good Clinical Practice (GCP) is followed to ensure accurate, reliable, and consistent data collection.

### Data management

Primary data management activities, which include data entry and validation, data coding and cleaning, database quality control, disaster recovery plans, adverse event reporting and tracking systems, preparation and submission of safety and compliance reports, and preparation of final study database, are undertaken by the designated CRO. The on-site study data manager oversees data-related procedures at the study site and is supervised by the CRO data management staff. Data management activities are performed using Clindex® Clinical Trial and Data Management software, developed by Fortress Medical Systems. All data management activities are in compliance with International Council on Harmonization (ICH) GCP E6, regulatory, sponsoring organization, and institutional requirements for the protection of children and confidentiality of personal and health information.

### Outcomes

The primary endpoint is the proportion of children failing treatment by Day 6 (Table 1: treatment failure criteria). The secondary endpoints are proportions of children with adverse events and serious adverse events, clinical relapse (between Days 6 and 14 among children without treatment failure before or on Day 6), treatment failure by Day 6 or relapse by Day 14 among all randomized children, treatment failure among those with malaria at baseline, treatment failure among those with wheeze during screening, treatment failure among those with oxygen saturation (SpO_2_) < 93% in room air by pulse oximetry at baseline, treatment failure among those with moderate malnutrition (mid-upper arm circumference (MUAC) 11.5–13.5 cm), treatment failure among those with very fast breathing for age (> 70 breaths per minute for 2–11 months; > 60 breaths per minute for 12–59 months), and treatment failure by age group.

### Statistical analysis

Primary analyses will be performed based on the intent-to-treat principle using linear regression adjusted for age groups, study phase, and gender; sequential monitoring; and robust standard errors based on the Huber-White sandwich estimator [8, 9]. Additional sensitivity analyses will be performed using multiple imputations [10] and tipping point analyses. Analyses of secondary endpoints will use robust standard errors as well, but will not be adjusted for sequential monitoring or any other factors. Further details are available in the statistical analysis plan (Additional file 2: Appendix 2).

## Ethics and dissemination

### Ethical approval and consent

The study is done in accordance with the ICH GCP and the Declaration of Helsinki 2008. The study was approved by the Western Institutional Review Board in the state of Washington, USA; the Malawi College of Medicine Research and Ethics Committee, Blantyre, Malawi; and the Malawi Pharmacy, Medicines and Poisons Board. The investigators will obtain approval from the Malawi College of Medicine Research and Ethics Committee, Blantyre, Malawi for all subsequent protocol amendments and changes to the informed consent documents. Once ethical approval is obtained, the principal investigator will promptly communicate to the study site and distribute applicable protocol amendments and revised informed consent documents. Written informed consent is obtained by trained study staff from all eligible children’s caregivers prior to enrollment.

### Patient safety

There is close safety monitoring of all children participating in the study. For the first 2 days, children are hospitalized in a dedicated ward away from other sick children to minimize risk of nosocomial infection and to ensure initial continuous monitoring. Each participating child is evaluated by a study clinician at each study visit. If a child misses a study visit, home visits are conducted by trained study staff to ensure clinical evaluation. Every effort is made to trace all children in the study for the final outcome assessment. An emergency number is provided to all participants’ caregivers so that an on-call clinician can be reached at any time during study participation. As needed, children in the study may be evaluated at interim visits and/or referred for additional care.

All adverse events are assessed and managed in accordance with standard clinical practice at KCH, and documented by the study clinical team. Children with an adverse event are followed and treated until the adverse event is resolved or stabilized. All serious adverse events are reported to the principal investigator and a study safety medical officer for review within 24 h of the event. Serious adverse events are regularly reviewed by the CRO safety monitor and medical expert and compiled into reports for the co-investigators.

An independent DSMB reviews cumulative safety and study conduct data through two formal interim analyses. At a minimum, safety data presented to the DSMB includes summaries of data on adverse events, serious adverse events, adherence rates, treatment failure, and clinical relapse. The DSMB includes one local pediatrician, one pneumonia expert, and one biostatistician. The DSMB will consider recommending to stop the trial prior to maximum enrollment if they determine early non-inferiority, early inferiority, or there are safety concerns.

### Possible risks

There are several potential risks to study participation. This is a randomized trial that is investigating the appropriate duration of treatment with oral amoxicillin DT for chest indrawing pneumonia. It is possible that 3 days and 5 days of amoxicillin treatment are not equivalent for managing chest indrawing pneumonia and that children receiving the shorter course of antibiotics could suffer a higher treatment failure rate, with an increased risk of adverse events, re-hospitalization, or death. On the other hand, those children in the 5 day amoxicillin DT group may have received antibiotics that were unnecessary, increasing their exposure to antibiotics and the potential risks of medication side effects. In order to minimize the risk of adverse events, hospitalization, and death, eligibility criteria for this study have been carefully selected and a robust safety monitoring scheme is in place. The children with pneumonia most at risk for treatment failure and/or death will be excluded from this study, including those with WHO Integrated Management of Childhood Illness (IMCI) general and respiratory danger signs, severe acute malnutrition, and HIV infection or exposure. Safety monitoring for this study includes frequent clinical examination at study visits for the first 4 days, outcome assessment and a clinician on call via an emergency hotline for the first 14 days, treatment and tracking of all adverse events, and an external DSMB for review of cumulative safety and study conduct data.

Caregivers may feel compelled to enroll in the study in order to receive care for their child within a research setting, which may be perceived as of a higher quality than the standard of care. In order to minimize the risk of coercion, study staff will not be recruiting participants directly. Instead, hospital clinicians will inform caregivers about the study and refer only those who are interested. During the informed consent process, study staff will emphasize that the child will receive medical care whether enrolled in the study or not. Another possible risk involves blood specimen sampling at screening which can cause pain and bruising at or around the blood draw site. To mitigate this risk, all study staff who will be collecting specimens from children in the study will be trained in the appropriate procedures and supervised accordingly. Participation in the study has the potential to compromise care for hospitalized children if study procedures are prioritized above urgent clinical care for acute infections. In order to minimize the possibility that participation in this trial will interfere with medical management, study staff will have the primary responsibility for the clinical management of hospitalized children in accordance with standard procedures. Furthermore, recognizing that some children may not come back for the follow-up visits, we provide for trained study staff to locate children who miss their follow-up appointments and conduct these visits in the home. We also ensure that study staff take the time to educate caregivers on the importance of adhering to the treatment regimen and follow-up.

### Dissemination

We plan to disseminate study results in peer-reviewed journals and international conferences, targeting those involved in the clinical care of children in low-resource settings as well as those who develop and advise on policies and guidelines in those settings. The trial is registered with ClinicalTrials.gov number NCT02678195.

## Discussion

The following discussion outlines our efforts to safely and efficiently conduct a study that maintains the rigors of a double blind randomized controlled trial protocol with the goal of informative and generalizable results applicable to real world, non-study settings in African low-middle income countries.

### Efforts toward generalizable results

The study was carefully developed and pragmatically designed with inclusion and exclusion criteria to allow as generalizable results as possible without putting children with severe illness at risk. Children enrolled in this study are diagnosed with chest indrawing pneumonia based on WHO IMCI clinical guidance. While microbiological and/or radiological diagnosis may add improved specificity to the clinical diagnosis of pneumonia, the majority of low-resource settings do not have access to this testing, and children are typically diagnosed based on clinical criteria alone. Children with moderate malnutrition and malaria are included, whereas children with severe forms of acute malnutrition, anemia, malaria, or pneumonia are not. We are enrolling children with more severe pneumonia and/or underlying comorbidities (e.g., HIV infection of exposure) excluded from this chest indrawing pneumonia study into a parallel prospective observational study to provide additional evidence on the standard of care and outcomes for children with pneumonia, and to generate data on the generalizability of this chest indrawing study. Children in the observational study will receive treatment as per standard of care and will be followed by study staff with scheduled visits at Days 2 (if hospitalized), 4, 6 and 14. Study staff will conduct a phone follow-up at Day 30 for a final outcome assessment.

### Efforts towards rigorous protocol

Placebo and amoxicillin DT identical in appearance, smell, taste, and dispersion properties are used to ensure blinding. Dedicated trained study staff care and follow children enrolled in the trial to assure the protocol and standard operating procedures are followed, data are accurately collected, and the highest level of safety provided. Standardized training, supervision, oversight, and testing are undertaken to ensure quality, consistency, and harmonized trial procedures and implementation. In addition to regular site monitoring visits by Save the Children, a CRO is employed to assist with close external monitoring of the study which includes regular site monitoring visits to assess compliance with human subjects and other research regulations and guidelines, adherence to the study protocol and procedures, quality and accuracy of data collected, and quality of care and child safety.

### Limitations and bias

Limitations to this study and potential sources of bias include study drug non-adherence and loss to follow-up. To minimize non-adherence to the study drug, demonstrations of study drug administration are conducted and caregivers are provided with clear verbal, written, and pictogram instructions. Caregivers are also asked about pill counts at every visit to assess their self-reported adherence to the study drug, any other medications they may be giving their child, or any other health care they have sought. To minimize loss to follow-up, caregivers are provided with clear follow-up instructions as well as called the afternoon before their visits to remind them to come the following day. A travel stipend for all follow-up visits is also provided. In addition, children are followed up with home visits upon a missed visit. A potential source of bias may be caregiver/child suspicion of study group (amoxicillin vs placebo). To mitigate caregiver unblinding, in addition to ensuring that the double-blind is maintained, the study drug is tested to confirm amoxicillin and placebo are identical.

Other limitations to this study are the clinical diagnosis of pneumonia, rather than a microbiological or radiological one, and the limited follow-up duration of 14 days. All study staff receive rigorous training in the WHO IMCI classification of pneumonia; however, no microbiological or radiological tests are routinely undertaken unless clinically indicated. Of note, this was a pragmatic design decision as in standard clinical care in this setting, microbiological or radiological tests are not typically undertaken unless clinically indicated. In addition, it is possible that we are missing longer-term consequences post-treatment given the short period of follow-up of 14 days.

## Additional files

Additional file 1: Appendix 3. Case report forms. (PDF 518 kb)

Additional file 2: Appendix 1. Informed consent form for enrollment. (DOC 67 kb)

Additional file 3: Appendix 2. Study protocol and statistical analysis plan. (DOC 636 kb)

## Abbreviations

BDH: Bwaila District Hospital
CRO: Contract research organization
DSMB: Data safety monitoring board
DT: Dispersible tablets
GCP: Good clinical practice
ICH: International council on harmonisation
IMCI: Integrated management of childhood illness
KCH: Kamuzu Central Hospital
mRDT: Malaria rapid diagnostic test
MUAC: Mid-upper arm circumference
SpO2: Oxygen saturation
WHO: World Health Organization
