# Supplementary material for: Methods for conducting a double-blind randomized controlled clinical trial of three days versus five days of amoxicillin dispersible tablets for chest indrawing childhood pneumonia among children two to 59 months of age in Lilongwe, Malawi: a study protocol
Source: BMC Infect Dis. 2018 Sep 21;18:476. doi: 10.1186/s12879-018-3379-z (PMC6151015; doi:10.1186/s12879-018-3379-z)
Supplement: Supplementary file 1 — Appendix 3. Case report forms. (PDF 518 kb) [file 12879_2018_3379_MOESM1_ESM.pdf]

Save the Children.	ITIP 2	VISIT 2<br>ENROLLMENT	Page No<br>7

Participant Number T 2 – _ _ – _ _ _ _ _

Visit date DD/MMM/YYYY

Informed Consent

Staff initials: _ _ _

Date and time of informed consent:	DD/MMM/YYYY	HH:MM	1
Consented at:	1 KCH	2 Bwaila	2

Vaccination, Vitamin A and Deworming History

Staff initials: _ _ _

At Birth

	Yes	No	Unknown			
BCG:	1	2	99	Date:	DD/MMM/YYYY	3
OPV (0):	1	2	99	Date:	DD/MMM/YYYY	4

6 Weeks

	Yes	No	Unknown			
OPV (1):	1	2	99	Date:	DD/MMM/YYYY	5
Pentavalent (1):	1	2	99	Date:	DD/MMM/YYYY	6
Pneumococcal (1):	1	2	99	Date:	DD/MMM/YYYY	7
Rotavirus (1):	1	2	99	Date:	DD/MMM/YYYY	8

10 Weeks

66 Not Applicable 9

	Yes	No	Unknown			
OPV (2):	1	2	99	Date:	DD/MMM/YYYY	10
Pentavalent (2):	1	2	99	Date:	DD/MMM/YYYY	11
Pneumococcal (2):	1	2	99	Date:	DD/MMM/YYYY	12
Rotavirus (2):	1	2	99	Date:	DD/MMM/YYYY	13

14 Weeks

66 Not Applicable 14

	Yes	No	Unknown			
OPV (3):	1	2	99	Date:	DD/MMM/YYYY	15
Pentavalent (3):	1	2	99	Date:	DD/MMM/YYYY	16
Pneumococcal (3):	1	2	99	Date:	DD/MMM/YYYY	17

9 Months

66 Not Applicable 18

	Yes	No	Unknown			
Measles:	1	2	99	Date:	DD/MMM/YYYY	19

Additional Vaccinations

Any additional vaccination(s):	1 Yes	2 No	99 Unknown		20
Description:	____________	Date:	DD/MMM/YYYY		21
Description:	____________	Date:	DD/MMM/YYYY		22
Description:	____________	Date:	DD/MMM/YYYY		23
Vitamin A supplement given in last 6 months:	1 Yes	2 No	99 Unknown	66 Not applicable	24
Date of last vitamin A:	DD/MMM/YYYY				25
Deworming done in last 6 months:	1 Yes	2 No	99 Unknown	66 Not applicable	26
Date of last deworming:	DD/MMM/YYYY				27

Participant Number

T 2 – __ – ______

Socio-Demographics

Staff initials: ___

Age of mother:	__ years ***OR*** 99 Unknown			1
Mother`s highest level of education:	1 Primary<br>4 None	2 Secondary<br>99 Unknown	3 Tertiary	2
Father`s highest level of education:	1 Primary<br>4 None	2 Secondary<br>99 Unknown	3 Tertiary	3
Parents` monthly income:	MK______ ***OR*** 99 Unknown			4

Environmental Exposure

Staff initials: ___

How many children aged 0-10 years (including study child) live in the same household:	__ **OR** 99 Unknown			5
Does this child attend out of home care (nursery/preschool/family care/crèche)?	1 Yes	2 No	99 Unknown	6
Do any children in the home other than the child attend out of home care (nursery/preschool/family care/crèche)?	1 Yes	2 No	99 Unknown	7
Does anyone who lives in the same household as the child smoke cigarettes?	1 Yes	2 No	99 Unknown	8
What is the main source of drinking water?	1 Piped water (inside)<br>3 Tube/Deep tube well<br>5 Surface water*<br>55 Other; specify: __________<br>99 Unknown	2 Piped water (outside)<br>4 Surface well/other well<br>6 Bottled/filtered water		9
In the last 24 hours, have you used soap and water to wash your hands?	1 Yes	2 No		10
Does this child sleep under a mosquito net?	1 Always<br>4 Never	2 Usually<br>99 Unknown	3 Sometimes	11
Is there cooking/heating that produces smoke in the household?	1 Yes	2 No	99 Unknown	12
Is anyone with active tuberculosis disease residing in or visiting the same household as the child?	1 Yes	2 No	99 Unknown	13

* Pond/tank/lake/ river/stream

Save the Children.	ITIP 2	VISIT 2<br>ENROLLMENT	Page No<br>9

Participant Number

T 2 – ⎿⎿ – ⎿⎿⎿⎿⎿

Respondent Assessment

Staff initials: ⎿⎿⎿

Fast/difficult breathing:	1 Yes	2 No	99 Unknown	1
Chest-indrawing:	1 Yes	2 No	99 Unknown	2
Lethargy:	1 Yes	2 No	99 Unknown	3
Feeling cold to the touch:	1 Yes	2 No	99 Unknown	4
Poor feeding:	1 Yes	2 No	99 Unknown	5
Vomiting:	1 Yes	2 No	99 Unknown	6
Diarrhoea:	1 Yes	2 No	99 Unknown	7
If Yes; how many episodes:	⎿⎿ in ⎿⎿ days			8
Nasal blockage:	1 Yes	2 No	99 Unknown	9
Runny nose:	1 Yes	2 No	99 Unknown	10
Measles in the last 3 months:	1 Yes	2 No	99 Unknown	11
Currently breastfeeding:	1 Yes	2 No	99 Unknown	12
If Yes; exclusive breastfeeding:	1 Yes<br>2 No, complementary feeding started at ⎿⎿ months<br>99 Unknown	66 Not applicable		13
If No; was the child breastfed:	1 Yes, complementary feeding started at ⎿⎿ months<br>2 No<br>99 Unknown	66 Not applicable		14

Physical Examination

Staff initials: ⎿⎿⎿

Time of examination	H H : M M			15
Respiratory rate:	⎿⎿⎿ breaths/min	Oxygen saturation	⎿⎿⎿ %	16
Pulse rate:	⎿⎿⎿ beats/min	Axillary temperature:	⎿⎿.⎿ °C	17

Interviewer Details

Interviewer signature:	____________________	Date:	D D / M M M / Y Y Y Y	18
Staff initials:	⎿⎿⎿			19

Save the Children.

ITIP 2	VISIT 3	Page No
	Day 2	10

Participant Number T 2 - __ __ - __ __ __ __ __

Visit date D D / M M M / Y Y Y Y

General Information

Staff initials: __ __ __

Start time of visit:	H H : M M	1
Follow-up location:	1 KCH inpatient 2 KCH outpatient 3 Home<br>55 Other; please specify: ____________	2
Relationship of respondent:	1 Mother 2 Father<br>55 Other; please specify: ____________	3
Adult respondent's statement - "Since the last visit the child is":	1 Improving 2 Not changed 3 Deteriorating<br>99 Unknown	4

Physical Examination

Staff initials: __ __ __

Respiratory rate:	__ __ __ breaths/min	Oxygen saturation	__ __ __ %	5
Pulse rate:	__ __ __ beats/min	Axillary temperature:	__ __ . __ °C	6

Respondent Assessment

Staff initials: __ __ __

Since the last visit, did the child have the following symptoms?

Fever:	1 Yes	2 No	99 Unknown	7
Cough:	1 Yes	2 No	99 Unknown	8
Fast/difficult breathing:	1 Yes	2 No	99 Unknown	9
Chest-indrawing:	1 Yes	2 No	99 Unknown	10
Lethargy:	1 Yes	2 No	99 Unknown	11
Feeling cold to the touch:	1 Yes	2 No	99 Unknown	12
Poor feeding:	1 Yes	2 No	99 Unknown	13
Vomiting:	1 Yes	2 No	99 Unknown	14
Diarrhoea:	1 Yes	2 No	99 Unknown	15
If Yes; how many episodes:	__ __ in __ __ days			16
Nasal blockage:	1 Yes	2 No	99 Unknown	17
Runny nose:	1 Yes	2 No	99 Unknown	18

Save the Children	ITIP 2	VISIT 3	Page No
		Day 2	11

Participant Number T 2 – __ __ – __ __ __ __ __

Clinician Assessment

Staff initials: __ __ __

Does the child currently have the following symptoms?

Cough:	1 Yes	2 No	99 Unknown	2
Fast/difficult breathing:	1 Yes	2 No	99 Unknown	3
Chest-indrawing:	1 Yes	2 No	99 Unknown	4
Feeling cold to the touch*:	1 Yes	2 No	99 Unknown	5
Signs of dehydration*:	1 Yes	2 No	99 Unknown	6
Poor feeding:	1 Yes	2 No	99 Unknown	7
Diarrhoea:	1 Yes	2 No	99 Unknown	8
Nasal blockage:	1 Yes	2 No	99 Unknown	9
Runny nose:	1 Yes	2 No	99 Unknown	10

* If not currently hospitalized, please refer to hospital for further evaluation

Danger Signs and Treatment Failure Criteria

Staff initials: __ __ __

Danger Signs*

Convulsions:	1 No convulsion<br>3 Observed convulsion (now)	2 Reported convulsion (within 24 hours)	11
Lethargic or unconscious:	1 Yes	2 No	12
Unable to drink or feed:	1 Yes	2 No	13
Vomits everything:	1 Yes	2 No	14
Stridor in calm child:	1 Yes	2 No	15

Respiratory Distress*

Grunting:	1 Yes	2 No	16
Nasal flaring:	1 Yes	2 No	17
Head nodding:	1 Yes	2 No	18
Severe chest-indrawing:	1 Yes	2 No	19
Other:	1 Yes	2 No	20
If Other; specify:	____________________		21

* If not currently hospitalized, please refer to hospital for further evaluation

Save the Children.	ITIP 2	VISIT 3<br>Day 2	Page No<br>12

Participant Number

T 2 – ⎵⎵ – ⎵⎵⎵⎵⎵

Danger Signs and Treatment Failure Criteria (Continued)

Other

Hypoxia (SaO_2 <90%):	1 Yes	2 No	1
Missing 3 or more doses due to vomiting:	1 Yes	2 No	2
Change in antibiotics:	1 Yes	2 No	3
Death:	1 Yes	2 No	4

Referral to Hospital

Staff initials: ⎵⎵⎵

If child is not in the hospital and required referral, was child referred?	1 Yes	2 No	3 In hospital	66 Not applicable	5
If No; specify:	______________________				6

Interviewer Details

Interviewer signature:	______________________	Date: D D / M M M / Y Y Y Y	7
Staff initials:	⎵⎵⎵		8

Participant Number T 2 – __ – ____

Visit date DD/MMM/YYYY

General Information

Staff initials: ___

Start time of visit:	HH:MM	1
Follow-up location:	1 KCH inpatient; 2 KCH outpatient; 3 Home; 55 Other; please specify: ______	2
Relationship of respondent:	1 Mother; 2 Father; 55 Other; please specify: ______	3
Adult respondent's statement - "Since the last visit the child is":	1 Improving; 2 Not changed; 3 Deteriorating; 99 Unknown	4

Physical Examination

Staff initials: ___

Respiratory rate:	___ breaths/min	Oxygen saturation	___ %	5
Pulse rate:	___ beats/min	Axillary temperature:	__._ °C	6

Respondent Assessment

Staff initials: ___

Since the last visit, did the child have the following symptoms?

Fever:	1 Yes	2 No	99 Unknown	7
Cough:	1 Yes	2 No	99 Unknown	8
Fast/difficult breathing:	1 Yes	2 No	99 Unknown	9
Chest-indrawing:	1 Yes	2 No	99 Unknown	10
Lethargy:	1 Yes	2 No	99 Unknown	11
Feeling cold to the touch:	1 Yes	2 No	99 Unknown	12
Poor feeding:	1 Yes	2 No	99 Unknown	13
Vomiting:	1 Yes	2 No	99 Unknown	14
Diarrhoea:	1 Yes	2 No	99 Unknown	15
If Yes; how many episodes:	__ in __ days			16
Nasal blockage:	1 Yes	2 No	99 Unknown	17
Runny nose:	1 Yes	2 No	99 Unknown	18

Save the Children.

ITIP 2	VISIT 4<br>DAY 4	Page No<br>14

Participant Number

T 2 – __ __ – __ __ __ __ __

Clinician Assessment

Staff initials: __ __ __

Does the child currently have the following symptoms?

Cough:	1 Yes	2 No	99 Unknown	2
Fast/difficult breathing:	1 Yes	2 No	99 Unknown	3
Chest-indrawing:	1 Yes	2 No	99 Unknown	4
Feeling cold to the touch:	1 Yes	2 No	99 Unknown	5
Signs of dehydration:	1 Yes	2 No	99 Unknown	6
Poor feeding:	1 Yes	2 No	99 Unknown	7
Diarrhoea:	1 Yes	2 No	99 Unknown	8
Nasal blockage:	1 Yes	2 No	99 Unknown	9
Runny nose:	1 Yes	2 No	99 Unknown	10

Danger Signs and Treatment Failure Criteria

Staff initials: __ __ __

Danger Signs*

Convulsions:	1 No convulsion<br>3 Observed convulsion (now)	2 Reported convulsion (within 24 hours)	11
Lethargic or unconscious:	1 Yes	2 No	12
Unable to drink or feed:	1 Yes	2 No	13
Vomits everything:	1 Yes	2 No	14
Stridor in calm child:	1 Yes	2 No	15

Respiratory Distress*

Grunting:	1 Yes	2 No	16
Nasal flaring:	1 Yes	2 No	17
Head nodding:	1 Yes	2 No	18
Severe chest-indrawing:	1 Yes	2 No	19
Other:	1 Yes	2 No	20
If Other; specify:	______________________		21

* If not currently hospitalized, please refer to hospital for further evaluation

Save the Children.	ITIP 2	VISIT 4<br>DAY 4	Page No<br>15

Participant Number

T 2 – __ __ – __ __ __ __ __

Danger Signs and Treatment Failure Criteria (Continued)

Other

Hypoxia (SaO_2 <90%):	1 Yes	2 No	1
Missing 3 or more doses due to vomiting:	1 Yes	2 No	2
Change in antibiotics:	1 Yes	2 No	3
Documented axillary temperature ≥ 38 °C with chest-indrawing:	1 Yes	2 No	4
Death:	1 Yes	2 No	5

Referral to Hospital

Staff initials: __ __ __

If child is not in the hospital and required referral, was child referred?	1 Yes	2 No	3 In hospital	66 Not applicable	6
If No; specify: ____________________					7

Interviewer Details

Interviewer signature: ____________________	Date: D D / M M M / Y Y Y Y	8
Staff initials: __ __ __		9

Save the Children	ITIP 2	VISIT 5<br>DAY 6	Page No<br>16

Participant Number T 2 - __ - ____ **Visit date** DD/MMM/YYYY

General Information

Staff initials: ___

Start time of visit:	HH:MM	1
Follow-up location:	1 KCH inpatient; 2 KCH outpatient; 3 Home; 55 Other; please specify: ____	2
Relationship of respondent:	1 Mother; 2 Father; 55 Other; please specify: ____	3
Adult respondent's statement - "Since the last visit the child is":	1 Improving; 2 Not changed; 3 Deteriorating; 99 Unknown	4

Physical Examination

Staff initials: ___

Respiratory rate:	___ breaths/min	Oxygen saturation	___ %	5
Pulse rate:	___ beats/min	Axillary temperature:	__._ °C	6

Respondent Assessment

Staff initials: ___

Since the last visit, did the child have the following symptoms?

Fever:	1 Yes	2 No	99 Unknown	7
Cough:	1 Yes	2 No	99 Unknown	8
Fast/difficult breathing:	1 Yes	2 No	99 Unknown	9
Chest-indrawing:	1 Yes	2 No	99 Unknown	10
Lethargy:	1 Yes	2 No	99 Unknown	11
Feeling cold to the touch:	1 Yes	2 No	99 Unknown	12
Poor feeding:	1 Yes	2 No	99 Unknown	13
Vomiting:	1 Yes	2 No	99 Unknown	14
Diarrhoea:	1 Yes	2 No	99 Unknown	15
If Yes; how many episodes:	__ in __ days			16
Nasal blockage:	1 Yes	2 No	99 Unknown	17
Runny nose:	1 Yes	2 No	99 Unknown	18

Save the Children	ITIP 2	VISIT 5<br>DAY 6	Page No<br>17

Participant Number
T 2 – ⌊ ⌊ ⌋ – ⌊ ⌊ ⌊ ⌊ ⌋

Clinician Assessment

Staff initials: ⌊ ⌊ ⌋

Does the child currently have the following symptoms?

Cough:	1 Yes	2 No	99 Unknown	2
Fast/difficult breathing:	1 Yes	2 No	99 Unknown	3
Chest-indrawing*:	1 Yes	2 No	99 Unknown	4
Feeling cold to the touch:	1 Yes	2 No	99 Unknown	5
Signs of dehydration:	1 Yes	2 No	99 Unknown	6
Poor feeding:	1 Yes	2 No	99 Unknown	7
Diarrhoea:	1 Yes	2 No	99 Unknown	8
Nasal blockage:	1 Yes	2 No	99 Unknown	9
Runny nose:	1 Yes	2 No	99 Unknown	10

* If not currently hospitalized, please refer to hospital for further evaluation

Danger Signs and Treatment Failure Criteria

Staff initials: ⌊ ⌊ ⌋

Danger Signs*

Convulsions:	1 No convulsion<br>3 Observed convulsion (now)	2 Reported convulsion (within 24 hours)	11
Lethargic or unconscious:	1 Yes	2 No	12
Unable to drink or feed:	1 Yes	2 No	13
Vomits everything:	1 Yes	2 No	14
Stridor in calm child:	1 Yes	2 No	15

Respiratory Distress*

Grunting:	1 Yes	2 No	16
Nasal flaring:	1 Yes	2 No	17
Head nodding:	1 Yes	2 No	18
Severe chest-indrawing:	1 Yes	2 No	19
Other:	1 Yes	2 No	20
If Other; specify:	______________________		21

* If not currently hospitalized, please refer to hospital for further evaluation

Danger Signs and Treatment Failure Criteria (Continued)

Other

Hypoxia (SaO_2 <90%):	1 Yes	2 No	1
Missing 3 or more doses due to vomiting:	1 Yes	2 No	2
Change in antibiotics:	1 Yes	2 No	3
Persistence of chest-indrawing:	1 Yes	2 No	4
Axillary temperature > 38 ºC:	1 Yes	2 No	5
Death:	1 Yes	2 No	6

Referral to Hospital

Staff initials: ___

If child is not in the hospital and required referral, was child referred?	1 Yes	2 No	3 In hospital	66 Not applicable	7
If No; specify: ______________________					8

Interviewer Details

Interviewer signature: ______________________ Date: D D / M M M / Y Y Y Y 9

Staff initials: ___ 10

Save the Children.	ITIP 2	VISIT 6<br>DAY 14	Page No<br>19

Participant Number

T 2 – __ __ – __ __ __ __ __

Visit date

D D / M M M / Y Y Y Y

General Information

Staff initials: ___ ___ ___

Start time of visit:	H H : M M	1
Follow-up location:	1 KCH inpatient 2 KCH outpatient 3 Home<br>55 Other; please specify: ____________	2
Relationship of respondent:	1 Mother 2 Father<br>55 Other; please specify: ____________	3
Adult respondent's statement - "Since the last visit the child is":	1 Improving 2 Not changed 3 Deteriorating<br>99 Unknown	4

Physical Examination

Staff initials: ___ ___ ___

Respiratory rate:	___ ___ ___ breaths/min	Oxygen saturation	___ ___ ___ %	5
Pulse rate:	___ ___ ___ beats/min	Axillary temperature:	___ ___ . ___ °C	6

Respondent Assessment

Staff initials: ___ ___ ___

Since the last visit, did the child have the following symptoms?

Fever:	1 Yes	2 No	99 Unknown	7
Cough:	1 Yes	2 No	99 Unknown	8
Fast/difficult breathing:	1 Yes	2 No	99 Unknown	9
Chest-indrawing:	1 Yes	2 No	99 Unknown	10
Lethargy:	1 Yes	2 No	99 Unknown	11
Feeling cold to the touch:	1 Yes	2 No	99 Unknown	12
Poor feeding:	1 Yes	2 No	99 Unknown	13
Vomiting:	1 Yes	2 No	99 Unknown	14
Diarrhoea:	1 Yes	2 No	99 Unknown	15
If Yes; how many episodes:	___ ___ in ___ ___ days			16
Nasal blockage:	1 Yes	2 No	99 Unknown	17
Runny nose:	1 Yes	2 No	99 Unknown	18

Save the Children	ITIP 2	VISIT 6<br>DAY 14	Page No<br>20

Participant Number
T 2 – __ __ – __ __ __ __ __

Clinician Assessment

Staff initials: __ __ __

Does the child currently have the following symptoms?

Cough:	1 Yes	2 No	99 Unknown	2
Fast/difficult breathing:	1 Yes	2 No	99 Unknown	3
Chest-indrawing*:	1 Yes	2 No	99 Unknown	4
Feeling cold to the touch:	1 Yes	2 No	99 Unknown	5
Signs of dehydration:	1 Yes	2 No	99 Unknown	6
Poor feeding:	1 Yes	2 No	99 Unknown	7
Diarrhoea:	1 Yes	2 No	99 Unknown	8
Nasal blockage:	1 Yes	2 No	99 Unknown	9
Runny nose:	1 Yes	2 No	99 Unknown	10

* If not currently hospitalized, please refer to hospital for further evaluation

Clinical Relapse Criteria

Staff initials: __ __ __

Danger Signs*

Convulsions:	1 No convulsion<br>3 Observed convulsion (now)	2 Reported convulsion (within 24 hours)	11
Lethargic or unconscious:	1 Yes	2 No	12
Unable to drink or feed:	1 Yes	2 No	13
Vomits everything:	1 Yes	2 No	14

Respiratory Distress*

Grunting:	1 Yes	2 No	15
Nasal flaring:	1 Yes	2 No	16
Head nodding:	1 Yes	2 No	17
Severe chest-indrawing:	1 Yes	2 No	18
Other:	1 Yes	2 No	19
If Other; specify:	______________________		20

Save the Children.	ITIP 2	VISIT 6	Page No
		DAY 14	21

Participant Number

T 2 – _ _ – _ _ _ _ _

Clinical Relapse Criteria (Continued)

Other

Hypoxia (SaO_2 <90%):	1 Yes	2 No	1
Fever (Axillary temperature > 38 ºC):	1 Yes	2 No	2
Recurrence of signs of chest-indrawing pneumonia:	1 Yes	2 No	3
Other severe disease:	1 Yes	2 No	4
If Yes; specify:	____________________		5
Death:	1 Yes	2 No	6

* If not currently hospitalized, please refer to hospital for further evaluation

Referral to Hospital

Staff initials: _ _ _

If child is not in the hospital and required referral, was child referred?	1 Yes	2 No	3 In hospital	66 Not applicable	7
If No; specify:	____________________				8

Clinical Outcome

Staff initials: _ _ _

Clinical outcome after 14 days from diagnosis:	1 Clinically cured<br>1 Failed initial antibiotic treatment regimen<br>2 Did not fail initial antibiotic treatment regimen<br>2 Not cured<br>1 Improving<br>2 Deteriorating<br>3 Stable (Prognosis unclear)<br>55 Other; please specify: ____________	9

Interviewer Details

Interviewer signature:	____________________	Date:	D D / M M M / Y Y Y Y	10
Staff initials:	_ _ _			11

Study Product Log

Staff initials: ___

First dose administered by:	1 Study staff	2 KCH staff	3 Caregiver	1
First dose administered at:	HH:MM			2
Randomization number:	R__ – ____ – _	Pharmacist: ___	Verified by: ___	3
Number of tablets dispensed at enrolment:	__			4

Day	Treatment number	Dose **successfully**[$] administered: Yes	No	Unknown	Number of **doses**[#] attempted	Reason dose not administered:	Did the child vomit within 30 minutes of a dose?: Yes	No	Un-known	N/A	Number of **doses**[#] where child vomited within 30 minutes	Docu-mented by:	
1	1	1	2	99	☐ 66 N/A		1	2	99	66	☐ 66 N/A	___	5
	2	1	2	99	☐ 66 N/A		1	2	99	66	☐ 66 N/A	___	6
2	1	1	2	99	☐ 66 N/A		1	2	99	66	☐ 66 N/A	___	7
	2	1	2	99	☐ 66 N/A		1	2	99	66	☐ 66 N/A	___	8
3	1	1	2	99	☐ 66 N/A		1	2	99	66	☐ 66 N/A	___	9
	2	1	2	99	☐ 66 N/A		1	2	99	66	☐ 66 N/A	___	10
4	1	1	2	99	☐ 66 N/A		1	2	99	66	☐ 66 N/A	___	11
	2	1	2	99	☐ 66 N/A		1	2	99	66	☐ 66 N/A	___	12
5	1	1	2	99	☐ 66 N/A		1	2	99	66	☐ 66 N/A	___	13
	2	1	2	99	☐ 66 N/A		1	2	99	66	☐ 66 N/A	___	14

Number of replacement tablets dispensed:	__ on DD/MMM/YYYY		66 Not applicable	Pharmacist: ___	10
Number of replacement tablets dispensed:	__ on DD/MMM/YYYY		66 Not applicable	Pharmacist: ___	12
Number of tablets returned:	__	99 Unknown	66 Not applicable; Clinic staff: ___	Pharmacist: ___	13
All blister packs returned?	1 Yes	2 No			18
Reason why study product not returned:	______				19

[$] ***Successful administration includes doses that were swallowed, but where vomiting occurred afterwards***
[#] ***Please record the number of the doses, <u>NOT</u> the number of tablets***

Hospitalization Log

Staff initials: ___

No	Admission date and time	Discharge date and time	Reason for admission	* Specify	
1	D D / M M M / Y Y Y Y<br>H H : M M	D D / M M M / Y Y Y Y<br>H H : M M **OR** 1 Ongoing	1 Protocol specified 2 Severe pneumonia 3 Malaria<br>4 Severe diarrhoea 5 WHO danger sign* 55 Other*		1
2	D D / M M M / Y Y Y Y<br>H H : M M	D D / M M M / Y Y Y Y<br>H H : M M **OR** 1 Ongoing	1 Protocol specified 2 Severe pneumonia 3 Malaria<br>4 Severe diarrhoea 5 WHO danger sign* 55 Other*		2
3	D D / M M M / Y Y Y Y<br>H H : M M	D D / M M M / Y Y Y Y<br>H H : M M **OR** 1 Ongoing	1 Protocol specified 2 Severe pneumonia 3 Malaria<br>4 Severe diarrhoea 5 WHO danger sign* 55 Other*		3
4	D D / M M M / Y Y Y Y<br>H H : M M	D D / M M M / Y Y Y Y<br>H H : M M **OR** 1 Ongoing	1 Protocol specified 2 Severe pneumonia 3 Malaria<br>4 Severe diarrhoea 5 WHO danger sign* 55 Other*		4
5	D D / M M M / Y Y Y Y<br>H H : M M	D D / M M M / Y Y Y Y<br>H H : M M **OR** 1 Ongoing	1 Protocol specified 2 Severe pneumonia 3 Malaria<br>4 Severe diarrhoea 5 WHO danger sign* 55 Other*		5
6	D D / M M M / Y Y Y Y<br>H H : M M	D D / M M M / Y Y Y Y<br>H H : M M **OR** 1 Ongoing	1 Protocol specified 2 Severe pneumonia 3 Malaria<br>4 Severe diarrhoea 5 WHO danger sign* 55 Other*		6
7	D D / M M M / Y Y Y Y<br>H H : M M	D D / M M M / Y Y Y Y<br>H H : M M **OR** 1 Ongoing	1 Protocol specified 2 Severe pneumonia 3 Malaria<br>4 Severe diarrhoea 5 WHO danger sign* 55 Other*		7
8	D D / M M M / Y Y Y Y<br>H H : M M	D D / M M M / Y Y Y Y<br>H H : M M **OR** 1 Ongoing	1 Protocol specified 2 Severe pneumonia 3 Malaria<br>4 Severe diarrhoea 5 WHO danger sign* 55 Other*		8
9	D D / M M M / Y Y Y Y<br>H H : M M	D D / M M M / Y Y Y Y<br>H H : M M **OR** 1 Ongoing	1 Protocol specified 2 Severe pneumonia 3 Malaria<br>4 Severe diarrhoea 5 WHO danger sign* 55 Other*		9
10	D D / M M M / Y Y Y Y<br>H H : M M	D D / M M M / Y Y Y Y<br>H H : M M **OR** 1 Ongoing	1 Protocol specified 2 Severe pneumonia 3 Malaria<br>4 Severe diarrhoea 5 WHO danger sign* 55 Other*		10

Adverse Events

Staff initials: _ _ _

Did the child experience any adverse events during the study? 1 Yes 2 No 1

If "Yes"; please record details of all adverse events below (using one line per event):

No	Adverse event description	Start Date	Stop Date	Outcome[a]	Therapy[b] *(List all that apply)*	Relation-ship[c]	DAIDS Grading[d]	Serious-ness[e]	
1		DD/MMM/YYYY	DD/MMM/YYYY **OR** 1 Ongoing[f]		*Specify:____________				2
2		DD/MMM/YYYY	DD/MMM/YYYY **OR** 1 Ongoing[f]		*Specify:____________				3
3		DD/MMM/YYYY	DD/MMM/YYYY **OR** 1 Ongoing[f]		*Specify:____________				4
4		DD/MMM/YYYY	DD/MMM/YYYY **OR** 1 Ongoing[f]		*Specify:____________				5

[a]**Outcome:**	[b]**Therapy:**	[c]**Relationship:**	[d]**DAIDS Grading:**	[e]**Seriousness:**
1. Resolved	1. None	1. Certain	0. Grade 0	1. Not serious
2. Continuing	2 Antibiotics	2. Probable	1. Grade 1	2. Life-threatening
3. Death	3. Drug, other than antibiotics	3. Possible	2. Grade 2	3. Persistent disability or incapacity
66. Not applicable	55. Other*	4. Probably not	3. Grade 3	4. Hospitalization or prolongation of hospitalization
99. Unknown	99. Unknown	5. Not related	4. Grade 4	5. Death
	(All that apply)	6. Not assessable	5. Grade 5	6. Congenital anomalies of birth defects

[f] Ongoing at study exit

Is this the last adverse events page? 1 Yes 2 No

Is this the last adverse events page? 1 Yes 2 No

Save the Children	ITIP 2	General Documents<br>Concomitant Antibiotics	Participant Number<br>T 2 – ⌊ ⌋ – ⌊ ⌋	Page No<br>**25.1**

Concomitant Antibiotics

Staff initials: ⌊ ⌋

Did the child take any concomitant antibiotics during the study period (from screening to the conclusion of participation)? If "Yes"; please record details below (using one line per medication): ☐1 Yes ☐2 No 1

No	Medication[a]	Formu-lation[c]	Total daily dose	Route[b]	Start Date	Stop Date	Indication	
1					D D / M M M / Y Y Y Y	D D / M M M / Y Y Y Y **OR** ☐1 Ongoing		2
2					D D / M M M / Y Y Y Y	D D / M M M / Y Y Y Y **OR** ☐1 Ongoing		3
3					D D / M M M / Y Y Y Y	D D / M M M / Y Y Y Y **OR** ☐1 Ongoing		4
4					D D / M M M / Y Y Y Y	D D / M M M / Y Y Y Y **OR** ☐1 Ongoing		5
5					D D / M M M / Y Y Y Y	D D / M M M / Y Y Y Y **OR** ☐1 Ongoing		6

[a] Please use generic names for all products with a single active ingredient, and trade names for all combination products.

[b] **Route of Administration:**

1.	Topical	4.	Intramuscular	7.	Intranasal	10.	Into conjunctival sac
2.	Oral	5.	Intravenous	8.	Inhaled	11.	Per vagina
3.	Per rectum.	6.	Subcutaneous	9.	Into auditory meatus	55.	Other

[c] **Formulation:**

1.	Tablet	4.	Injectable	7.	Drops	10.	Suppository
2.	Capsule	5.	Cream	8.	Spray	55.	Other
3.	Suspension/Syrup	6.	Gel	9.	Inhalation		

Is this the last concomitant antibiotics page? ☐1 Yes ☐2 No

Save the Children	ITIP 2	General Documents<br>Concomitant Medications	Participant Number<br>T 2 – __ __ – __ __ __ __ __	Page No<br>**26.1**

Prior and Concomitant Medications

Staff initials: __ __ __

Did the child take any medication prior to enrolment (48hrs) or any concomitant medication during the study period (from screening to the conclusion of participation)? ☐1 Yes ☐2 No 1

If "Yes"; please record details below (using one line per medication):

No	Medication[a]	Formu-lation[c]	Total daily dose	Route[b]	Start Date	Stop Date	Indication	
1					DD/MMM/YYYY	DD/MMM/YYYY<br>**OR** ☐1 Ongoing		2
2					DD/MMM/YYYY	DD/MMM/YYYY<br>**OR** ☐1 Ongoing		3
3					DD/MMM/YYYY	DD/MMM/YYYY<br>**OR** ☐1 Ongoing		4
4					DD/MMM/YYYY	DD/MMM/YYYY<br>**OR** ☐1 Ongoing		5
5					DD/MMM/YYYY	DD/MMM/YYYY<br>**OR** ☐1 Ongoing		6

[a] Please use generic names for all products with a single active ingredient, and trade names for all combination products.

[b] **Route of Administration:**

1.	Topical	4.	Intramuscular	7.	Intranasal	10.	Into conjunctival sac
2.	Oral	5.	Intravenous	8.	Inhaled	11.	Per vagina
3.	Per rectum.	6.	Subcutaneous	9.	Into auditory meatus	55.	Other

[c] **Formulation:**

1.	Tablet	4.	Injectable	7.	Drops	10.	Suppository
2.	Capsule	5.	Cream	8.	Spray	55.	Other
3.	Suspension/Syrup	6.	Gel	9.	Inhalation		

Is this the last concomitant medications page? ☐1 Yes ☐2 No

Save the Children.

ITIP 2	General Documents<br>Explanatory Notes & Missing Data	Participant Number<br>T 2 – __ __ – __ __ __ __ __	Page No<br>27.1

Explanatory Notes

66 Not applicable Staff initials: __ __ __ 1

No	Page	Comment	Initials	
1				2
2				3
3				4
4				5
5				6

Missing Data

66 Not applicable Staff initials: __ __ __ 7

No	Page	Missing data	Reason	Initials	
1					8
2					9
3					10
4					11
5					12

Is this the last explanatory notes and missing data page? 1 Yes 2 No

Conclusion of Participation

Staff initials: _ _ _

Date of Conclusion of Participation:	D D / M M M / Y Y Y Y		1
Child's status upon trial termination:	1 Completed	2 Drop-out	2
If "Drop-out", specify the primary reason for premature discontinuation from trial below (tick only one box):	1 Adverse event<br>3 Lost to follow-up<br>5 Consent withdrawn<br>55 Other, specify: ____________	2 Protocol non-compliance<br>4 Death<br>6 Sponsor decision	3
Was the child referred to continued care?	1 Yes	2 No	4
If Yes; describe referral:	____________		5
Is the participation in the study noted in the child's health passport?	1 Yes	2 No	6
If No; please comment:	____________		7

Checklist

Staff initials: _ _ _

Section		Number of pages/visits	
Unscheduled Visit (prior to Day 6)	66 Not applicable		8
Unscheduled Visit (post Day 6)	66 Not applicable		9
Additional Adverse Events	66 Not applicable		10
Additional Concomitant Antibiotics	66 Not applicable		11
Additional Concomitant Medications	66 Not applicable		12
Additional Explanatory Notes and/or Missing Data	66 Not applicable		13
Non-Compliance	66 Not applicable		14

Clinician Declaration

The information contained on this and the preceding pages, including the results of tests and evaluations performed, accurately reflects the medical records of this child.

Clinician signature: ____________ Date: D D / M M M / Y Y Y Y 15

Staff initials: _ _ _ 16
