# Supplementary material for: Methods for conducting a double-blind randomized controlled clinical trial of three days versus five days of amoxicillin dispersible tablets for chest indrawing childhood pneumonia among children two to 59 months of age in Lilongwe, Malawi: a study protocol
Source: BMC Infect Dis. 2018 Sep 21;18:476. doi: 10.1186/s12879-018-3379-z (PMC6151015; doi:10.1186/s12879-018-3379-z)
Supplement: Supplementary file 2 — Appendix 1. Informed consent form for enrollment. (DOC 67 kb) [file 12879_2018_3379_MOESM2_ESM.doc]

**Appendix 2. Informed consent form for enrollment**

**Innovative Treatments in Pneumonia – Chest-Indrawing Study (ITIP2)**

Version 7.0, January 12, 2016

**Protocol title:** Double-blind randomized controlled clinical trial of 3 days versus 5 days amoxicillin DT for chest-indrawing childhood pneumonia among children 2-59 months of age presenting to Kamuzu Central Hospital in Lilongwe, Malawi

**CO-LOCAL PRINCIPAL INVESTIGATORS:**

Dr. Ajib Phiri

Dr. Tisungane Mvalo

**Introduction**

You are being asked for your child to take part in this study because your child is between the ages of 2 and 59 months and your child has passed the screening questions and tests for the research study named above. This study is sponsored by Save the Children. The persons in charge of this study at this site are Dr. Ajib Phiri and Dr. Tisungane Mvalo. The enrollment process can include interview questions, rapid blood tests, and a physical exam

This is an enrollment consent form. It gives you information about the study product, study questions and exams, and what you and your child have to do for your child to be in the study. The study staff will explain the exams and tests to you and what is expected of you and your child. You are free to ask questions about the study at any time. If you agree for your child to take part in this study, you will be asked to sign this consent form or make your mark/thumbprint in front of a witness. You will be given a copy of this form to keep. Another copy will stay with the study records.

**Why is this Study Being Done?**

This study is being done to see if 3 days of antibiotics are as effective as 5 days of antibiotics for treating a child with chest-indrawing pneumonia. An antibiotic is medicine that prevents illness by helping your immune system fight bacterial infections. The antibiotic used in this study is called amoxicillin.

The Bill and Melinda Gates Foundation is providing funds for this study to take place. A total of 2,000 children from KCH will join this study. Each child will be in the study for a total of 14 days. If your child is in the study, you will have to attend 5 study visits with your child.

**What Do I Have To Do If I Take Part in the Study?**

If you agree for your child to be in the study, you will have these study visits here at the study site:

- Enrollment Visit (Day 1) - Today
- 2-Day Visit - Tomorrow
- 4-Day Visit
- 6-Day Visit
- 14-Day Visit – Two weeks from today

In between visits, you can come to the study clinic to be seen by study staff if your child has any side effects, worsening condition, other illness, or any other health problem. You can also call the phone number on this form to speak to someone on the study staff 24 hours a day. It is very important to let study staff know right away if anything does not seem right with your child’s health.

**Enrollment Visit**

The Enrollment Visit will continue today, after you read (or have read to you), discuss, and sign or make your mark/thumbprint on this form. No study activities will be started before they have been fully explained to you, you have let us know that you understand the enrollment process and you have signed or made your mark/thumbprint on this form.

The Enrollment visit will take about one hour. You and your child will be asked to do these things for the Enrollment Visit if you decide you want your child to be in the study:

- Sign this form or make your mark/thumbprint on it after you have read it (or have it read to you), understand it, and had the chance to ask questions about the study
- Tell the study staff how they can stay in contact with you, including the contact information for 2-3 people who can be contacted if the study staff is unable to reach you directly
- Tell the study staff about your family’s socio-demographics (like your education and income)
- Tell the study staff about any medical problems your child has had
- Tell the study staff about your child’s vaccinations and other medical history
- Tell the study staff about any medicines your child is taking now
- Have a physical exam for your child
- Your child will receive the first dose of study antibiotics
- Learn from study staff how to prepare the medicine to give to your child at home

Your child will be admitted to the hospital for 2 days so that study doctors and nurses may watch your child closely to make sure he or she is getting better on the antibiotics.

**Randomization**

If you agree for your child to take part in the study, your child will be randomly placed into one of two groups. This means that the way the decision is made is like tossing a coin. Your child has an equal chance of being placed in either group. You cannot choose a group for your child. The study staff also cannot choose a group for your child. We will use a computer to select the groups.

- GROUP 1 will be given antibiotics to take twice a day for 5 days.
- GROUP 2 will be given antibiotics to take twice a day for the first 3 days and placebo to take twice a day for the 2 days after that (5 days total). A placebo is fake medicine that looks and tastes like medicine but does not prevent any illness.

You will not know which group your child is in. The study doctors and nurses will also not know which group your child is in. Both groups will receive the same level of care and attention from doctors and nurses.

Both groups are very important to this study. We do not know if the approaches will be the same or different. We will find out if the approaches are the same by comparing the two groups of children.

**If you allow your child to take part in this study your child will have a 1 in 2 chance of receiving a shorter course of antibiotic than the approved standard course. We do not know whether this shorter course will be an effective treatment for pneumonia.**

The two groups will be compared every 6 months by a Board of independent experts (called a Data Safety Monitoring Board or DSMB). The DSMB will compare the groups to see if there are differences in how effective the treatments are. If there are benefits in one group, we will stop assigning children to groups.

**Alternative to study participation**

The current standard of care in Malawi for your child’s pneumonia diagnosis is to receive oral amoxicillin twice a day for 5 days. If you decide not to have your child participate in this research study, your child will receive the standard of care here at KCH.

**Study Product**

The tablets you will be given by the study staff to give to your child for the first 3 days will all be an antibiotic called amoxicillin. Amoxicillin is an approved medicine in Malawi that is regularly prescribed for pneumonia. The tablets you will receive to give to your child for the 2 days after that will either be placebo or an antibiotic. It is very important that you give your child all of the tablets in the package for the first 3 days before you give your child any of the tablets in the package for the next 2 days. The pills will be labeled for you.

The tablets dissolve in liquid to make it easy for your child to take. Put the tablets in 2-3 teaspoons of clean water or breast milk and wait for at least 1 minute before the tablet is completely dissolved before giving to your child to drink. Clean water should be filtered, boiled, or bottled.

Your child will need to take the tablets twice a day for 5 days. If your child is under 12 months of age, you will need to give your child 1 tablet each time. If your child is between 12 months and 3 years of age, you will need to give your child 2 tablets each time. If you child is between 3 years and 5 years of age, you will need to give your child 3 tablets each time.

**Follow-up visits**

Study staff will call you to remind you of your follow-up visits. These visits will take about an hour each. At these visits, you will:

- Bring all the study medicine packages you initially received to show the study staff
- Tell the study staff how they can stay in contact with you (let them know about any changes to your address, phone number or other ways to contact you)
- Tell the study staff about any medical problems your child has had since the last visit
- Have a physical exam for your child
- Tell the study staff how much of the medicine your child has taken since the last visit
- Review with study staff how to prepare the medicine to give to your child at home

**Home visits**

If you miss one of the 4 scheduled follow-up visits, study staff will try to contact you by phone to see if you can come back to the study clinic for the visit. If you cannot bring your child to the study site for the visit or the study staff is not able to reach you by phone, then study staff will visit your home to conduct the study visit. Home visits will have all of the same procedures as regular follow-up visits. If study staff are unable to find you at home, they may try to reach the contacts you listed for the study. Study staff will not tell other people that your child is in a study and will protect your privacy as much as possible.

**Why Would The Doctor Take My Child Out of This Study Early?**

The study doctor may need to take your child out of the study early if:

- The study is cancelled by the U.S. Food and Drug Administration (U.S. FDA), Save the Children, the Ethics Committee, the Office for Human Research Protections (OHRP), the Malawi government or regulatory agency, or the Institutional Review Board (IRB).
- The Data Safety and Monitoring Board (DSMB) recommends that the study be stopped early.
- You are not able to keep appointments.
- Other reasons that may prevent you and/or your child from completing the study successfully.

**What Are the Risks of Being in the Study?**

The antibiotic may cause nausea, vomiting, diarrhea and possibly rash. Allergic reactions are possible and could be life threatening.

**Risk of Treatment Failure and Adverse Events:**

Your child could be placed in a group that is not as effective or as safe at treating pneumonia as the other group. If your child is randomized to the group that only gets 3 days of antibiotics, your child may not get better or may get worse after that and will have to be switched to different medicine. In either group, there is the risk of getting worse and not responding to treatment right away. There may be other risks we do not know about right now.

**Are There Benefits To Taking Part In This Study?**

Your child can receive amoxicillin outside the study. Your child will not receive any additional benefit from being in this study. This study is designed to help the researchers find out whether a shorter course of antibiotics is safe and effective treatment for pneumonia; which may benefit patients with pneumonia in the future.

**What About Confidentiality?**

Efforts will be made to keep your and your child’s personal information private. We cannot guarantee absolute confidentiality. If this study is published, your name and/or your child’s will not be used and you and/or your child will not be personally identified.

In order to make sure the study is being done properly, your records may be reviewed by:

- The U.S. FDA or Malawi Pharmacy, Medicine, and Poison Board
- Study staff and monitors
- Ethics Committee or IRB
- Save the Children

Your child study records will be kept at the clinic/hospital for at least five years after the study is completed. You may see your child’s records. If you decide to leave the study, information already collected from your child may still be used for the study.

**What Are The Costs To Me?**

There is no cost to you for the study visits.

**Will I Receive Any Payment?**

At the end of each study visit, you will receive money for your transport expenses to and from the study clinic. You will receive money in our local currency equivalent to US$5 for each scheduled study visit. At the end of the current visit, you will receive MK 100 worth of airtime for your choice of mobile phone carrier, AirTel or TNM. This airtime is provided for you to be able to phone the study team if you have any questions related to the study, or if you wish to inform the study team that the condition of your child has worsened.

**What Happens If My Child Is Injured?**

It is unlikely that your child will be injured as a result of being in this study. If your child is injured due to being in this study, your child will be given immediate treatment. Contact Dr. Ajib Phiri at or Dr. Tisungane Mvalo– they will tell you where your child can get treatment.

The sponsor has taken out insurance from ACE/Illinois Union Insurance Company in the event of a screening or study-related injury to your child. Save the Children will pay to treat any injury suffered as a result of participation in the trial.

**What Are My Child’s Rights As A Research Participant?**

Having your child take part in the study is completely up to you. It’s your choice. You may choose to have your child stop the study procedures at any time – there will be no penalty or loss of benefits to which you/your child are otherwise entitled. You and your child will be treated the same no matter what you decide. If you choose to not have your child be in the study, you and your child will not lose the benefit of services to which you would normally have at this clinic and there will be no penalty or loss of benefits to which you/your child are otherwise entitled.

We will tell you about new information from this or other studies that may affect your child’s health, welfare or willingness to stay in this study. If you want the results of the study, let the study staff know that you would like them.

A description of this clinical trial will be available on [http://www.ClinicalTrials.gov](http://www.ClinicalTrials.gov/), as required by U.S. Law. This Web site will not include information that can identify you and/or your child. At most, the Web site will include a summary of the results. You can search this Web site at any time.

The research study was reviewed and approved by the College of Medicine Research and Ethics Committee; the Western Institutional Review Board in Washington, USA; the University of North Carolina Institutional Review Board in Chapel Hill, North Carolina, USA; and the Malawi Pharmacy, Medicines and Poisons Board. This approval does not mean that the study is safe or that the committees approved your child’s participation.

**What Do I Do If I have Problems or Questions?**

For questions, concerns or complaints about the study or if your child has a research-related injury, you should contact:

- Dr. Ajib Phiri
- Dr. Tisungane Mvalo

For questions, concerns or complaints about the study or questions about your child’s rights as a research participant, contact:

- College of Medicine Research and Ethics (COMREC) Secretariat

or

- Western Institutional Review Board® (WIRB®)

WIRB is a group of people who perform independent review of research.

**SIGNATURE**

I have read the informed consent (or had it read and explained to me), and all my questions have been answered. I have let the study staff know that I understand and I agree for my child to take part in this study. My signature or mark/thumbprint below documents my consent.

Participant’s Name

(Parent/Guardian to print participating child’s name)

Parent/Guardian Parent/Guardian’s Signature and Date

(print name)

Study Staff Conducting Study Staff Signature and Date

Consent Discussion (print name)

Witness’ Name (print name) Witness’s Signature and Date

(As appropriate, if Parent/Guardian is illiterate)
