# Supplementary material for: Methods for conducting a double-blind randomized controlled clinical trial of three days versus five days of amoxicillin dispersible tablets for chest indrawing childhood pneumonia among children two to 59 months of age in Lilongwe, Malawi: a study protocol
Source: BMC Infect Dis. 2018 Sep 21;18:476. doi: 10.1186/s12879-018-3379-z (PMC6151015; doi:10.1186/s12879-018-3379-z)
Supplement: Supplementary file 3 — Appendix 2. Study protocol and statistical analysis plan. (DOC 636 kb) [file 12879_2018_3379_MOESM3_ESM.doc]

**Appendix 3. Study protocol and statistical analysis plan**

**Title: Innovative Treatments in Pneumonia (ITIP) 2**

**A Study of the Pneumonia Innovations Team**

**Subtitle: Double-blind randomized controlled clinical trial of 3 days versus 5 days amoxicillin DT for chest-indrawing childhood pneumonia among children 2-59 months of age presenting to Kamuzu Central Hospital in Lilongwe, Malawi**

**Sponsored by:**

**Save the Children Federation, Inc.**

**Version 8.0**

**March 14, 2016**

**Confidentiality Statement**

**This document is confidential and is to be distributed for review only to investigators, potential investigators, consultants, study staff, applicable regulatory authorities, and applicable independent ethics committees or institutional review boards. The contents of this document shall not be disclosed to others without written authorization from Save the Children (or others, as applicable), unless it is necessary to obtain informed consent from potential study participants.**

**Innovative Treatments in Pneumonia 2**

## TABLE OF CONTENTS

[TABLE OF CONTENTS 2](#__RefHeading___Toc507589224)

[ABBREVIATIONS AND ACRONYMS 5](#__RefHeading___Toc507589225)

[INVESTIGATORS 6](#__RefHeading___Toc507589226)

[EXECUTIVE SUMMARY 8](#__RefHeading___Toc507589227)

[PROTOCOL OUTLINE 9](#__RefHeading___Toc507589228)

[1 BACKGROUND AND INTRODUCTION 11](#__RefHeading___Toc507589229)

[2 RATIONALE 12](#__RefHeading___Toc507589230)

[3 STUDY HYPOTHESIS, OBJECTIVES AND ENDPOINTS 13](#__RefHeading___Toc507589231)

[• Study Hypothesis 13](#__RefHeading___Toc507589232)

[• Study Objectives 13](#__RefHeading___Toc507589233)

[• Study Endpoints 14](#__RefHeading___Toc507589234)

[4 METHODOLOGY 14](#__RefHeading___Toc507589235)

[4.1 STUDY DESIGN 14](#__RefHeading___Toc507589236)

[4.2 STUDY SITE 15](#__RefHeading___Toc507589237)

[4.3 STUDY POPULATION 15](#__RefHeading___Toc507589238)

[• Study Population Overview 15](#__RefHeading___Toc507589239)

[• Participant Eligibility 16](#__RefHeading___Toc507589240)

[• Inclusion Criteria 16](#__RefHeading___Toc507589241)

[• Exclusion Criteria 16](#__RefHeading___Toc507589242)

[4.4 STUDY PERIOD 17](#__RefHeading___Toc507589243)

[4.5 SAMPLE SIZE 18](#__RefHeading___Toc507589244)

[4.6 STUDY PROCEDURES 18](#__RefHeading___Toc507589245)

[• Recruitment 20](#__RefHeading___Toc507589246)

[• Screening 21](#__RefHeading___Toc507589247)

[• Informed Consent 21](#__RefHeading___Toc507589248)

[• Enrollment Visit 22](#__RefHeading___Toc507589249)

[• Randomization 23](#__RefHeading___Toc507589250)

[• Management of Study Participants During Hospitalization 23](#__RefHeading___Toc507589251)

[• Follow-Up Visits 24](#__RefHeading___Toc507589252)

[• Missed Visits 25](#__RefHeading___Toc507589253)

[• Interim Contacts and Visits 25](#__RefHeading___Toc507589254)

[• Therapy for Treatment Failure and Clinical Relapse 25](#__RefHeading___Toc507589255)

[• Withdrawal and Early Termination 25](#__RefHeading___Toc507589256)

[• Study Termination Visit 26](#__RefHeading___Toc507589257)

[• Biohazard Containment 26](#__RefHeading___Toc507589258)

[4.7 STUDY PRODUCTS 26](#__RefHeading___Toc507589259)

[• Presentation and Formulation 26](#__RefHeading___Toc507589260)

[• Preparation and Administration 27](#__RefHeading___Toc507589261)

[• Stability and Storage 28](#__RefHeading___Toc507589262)

[• Accountability and Disposal 28](#__RefHeading___Toc507589263)

[4.8 DATA COLLECTION 28](#__RefHeading___Toc507589264)

[• Case Report Forms 29](#__RefHeading___Toc507589265)

[• Source Documents 29](#__RefHeading___Toc507589266)

[4.9 DATA MANAGEMENT 29](#__RefHeading___Toc507589267)

[• Data Access 30](#__RefHeading___Toc507589268)

[• Data Storage 30](#__RefHeading___Toc507589269)

[• External Study Monitoring 30](#__RefHeading___Toc507589270)

[4.10 SAFETY ASSESSMENTS AND REPORTING 31](#__RefHeading___Toc507589271)

[• Safety Monitoring 31](#__RefHeading___Toc507589272)

[• Data Safety and Monitoring Board (DSMB) 31](#__RefHeading___Toc507589273)

[• Adverse Events 32](#__RefHeading___Toc507589274)

[• Serious Adverse Event 32](#__RefHeading___Toc507589275)

[• Adverse Event Relationship to Study Product 33](#__RefHeading___Toc507589276)

[• Grading Severity of Events 33](#__RefHeading___Toc507589277)

[• Safety Reporting 33](#__RefHeading___Toc507589278)

[• Study Discontinuation 34](#__RefHeading___Toc507589279)

[5 STATISTICAL DESIGN AND ANALYSIS 34](#__RefHeading___Toc507589280)

[5.1 DATA ANALYSIS 34](#__RefHeading___Toc507589281)

[• Overview and General Design 34](#__RefHeading___Toc507589282)

[• Randomization and Blinding Procedures 34](#__RefHeading___Toc507589283)

[• Objectives and Endpoints 35](#__RefHeading___Toc507589284)

[• Analytical Methodology for Interim Analyses 37](#__RefHeading___Toc507589285)

[• Analytical Methodology for Final Analyses 37](#__RefHeading___Toc507589286)

[5.2 RESULT PRESENTATION 38](#__RefHeading___Toc507589287)

[5.3 DISSEMINATION OF RESULTS 38](#__RefHeading___Toc507589288)

[6 ETHICAL CONSIDERATIONS AND CONSENT 38](#__RefHeading___Toc507589289)

[• Principles for Clinical Research 38](#__RefHeading___Toc507589290)

[• Institutional Review Boards (IRBs) and Independent Ethics Committees (IECs) 38](#__RefHeading___Toc507589291)

[• Informed Consent 39](#__RefHeading___Toc507589292)

[• Risks to Participants 39](#__RefHeading___Toc507589293)

[• Protection against Risks 40](#__RefHeading___Toc507589294)

[• Benefits to Participants 40](#__RefHeading___Toc507589295)

[• Participant Confidentiality 41](#__RefHeading___Toc507589296)

[• Participant Reimbursement 41](#__RefHeading___Toc507589297)

[• Storage of Specimens 41](#__RefHeading___Toc507589298)

[7 POSSIBLE CONSTRAINTS 42](#__RefHeading___Toc507589299)

[8 REQUIREMENTS AND TRAINING 42](#__RefHeading___Toc507589300)

[9 REFERENCES 42](#__RefHeading___Toc507589301)

[Protocol Appendices 44](#__RefHeading___Toc507589302)

[Protocol Appendix I: Schedule of study visits and evaluations 44](#__RefHeading___Toc507589303)

[ProtocolAppendix II: Sample collection and laboratory evaluations 44](#__RefHeading___Toc507589304)

[Protocol Appendix III: Study requirements and training 45](#__RefHeading___Toc507589305)

[Protocol Appendix IV: Study sensitization/recruitment script 47](#__RefHeading___Toc507589306)

**Innovative Treatments in Pneumonia 2**

## ABBREVIATIONS AND ACRONYMS

AE adverse events

AIDS Acquired Immunodeficiency Syndrome

BDH Bwaila District Hospital

BMGF Bill & Melinda Gates Foundation

CFR Code of Federal Regulations

CI confidence interval

COM College of Medicine

COMREC College of Medicine Research and Ethics Committee

CRF case report form

CRO contract research organization

DSMB Data and Safety Monitoring Board

DT dispersible tablets

GCP Good Clinical Practices

HIV Human Immunodeficiency Virus

ICF informed consent form

ICH International Conference on Harmonisation of Technical Requirements for Registration of Pharmaceuticals for Human Use

ID identification

IEC independent ethics committee

IMCI Integrated Management of Childhood Illness

IRB institutional review board

ITIP Innovative Treatments in Pneumonia

KCH Kamuzu Central Hospital

kg kilogram

mg milligram

LAR legally authorized representative

MOH Ministry of Health

mRDT malaria rapid diagnostic test

OPD outpatient department

OR odds ratio

PI principal investigator

RR risk ratio

SAE serious adverse event

SC Save the Children Federation, Inc.

SAP Statistical Analysis Plan

SOP standard operating procedure(s)

U.S. FDA United States Food and Drug Administration

UNC University of North Carolina

UW University of Washington

WHO World Health Organization

**Innovative Treatments in Pneumonia 2**

##
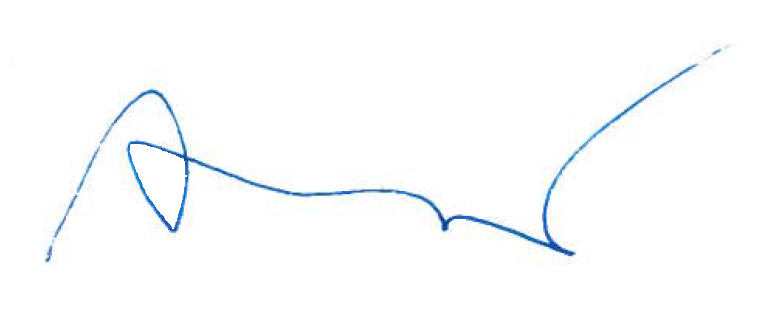
INVESTIGATORS

**Principal investigator (PI):**

Amy Ginsburg, MD, MPH ________________________03/14/2016

Senior Advisor, Child Health PI signature Date

Save the Children Federation, Inc.

**Local co-PIs:**

Ajib Phiri, MD

Senior Academic Staff, Department of Pediatrics and Child Health

Malawi College of Medicine

Tisungane Mvalo, MB BS, FC Paed

Consultant Pediatrician and Clinical Research Investigator

University of North Carolina (UNC) Project Lilongwe Trust, Malawi

**Co-investigators:**

Susanne May, PhD

Associate Professor, Department of Biostatistics

University of Washington

Norman Lufesi, CO, M.Phil.

Program Manager, Acute Respiratory Infection & Emergency Triage Assessment and Treatment

Malawi Ministry of Health

Eric McCollum, MD

Assistant Professor, Department of Pediatrics

Johns Hopkins School of Medicine

PARTICIPATING INSTITUTIONS

| **Sponsor:** | Save the Children Federation, Inc. (SC) | |
| --- | --- | --- |
| **Monitor:** | Triclinium Clinical Trial Project Management (Pty) Ltd. | |
| **Medical Officers:** | Amy Ginsburg, MD, MPH  Save the Children Federation, Inc. (SC) |  |
| **Clinical Trial Sites:** | Kamuzu Central Hospital | |
| **Data Center:** | Triclinium Clinical Trial Project Management (Pty) Ltd. | |
| **Study Operations:** | University of North Carolina (UNC) Project, Lilongwe Medical Relief Fund Trust | |
| **Statistical Support:** | University of Washington (UW) | |
| **Local Collaborators:** | Malawi Ministry of Health (MOH)  College of Medicine (COM) at the University of Malawi  Kamuzu Central Hospital (KCH)  Bwaila District Hospital (BDH)  Save the Children International – Malawi Country Office | |
| **Funding Agency:** | Bill and Melinda Gates Foundation (BMGF) | |

## EXECUTIVE SUMMARY

**Problem to be studied:** Pneumonia is responsible for more than one in five child deaths around the globe. Each year, approximately 1.1 million children die before their fifth birthdays due to pneumonia, more than the number of under-five deaths that result from human immunodeficiency virus (HIV), tuberculosis, and malaria combined. In addition to preventing pneumonia, there is a critical need to provide greater access to appropriate and effective treatment. Studies in Asia have evaluated the effectiveness of 3 days of oral amoxicillin for the treatment of fast-breathing pneumonia; however, further evidence is needed to determine if 3 days of oral amoxicillin is also effective for the treatment of chest-indrawing pneumonia. Finally, given the paucity of data from Africa, African-based research is necessary to establish optimal treatment regimens for childhood pneumonia in the region.

With the expressed support of the Malawi Ministry of Health (MOH) and in collaboration with external experts from the University of Washington (UW), Save the Children Federation, Inc. (SC) will work closely with investigators at the College of Medicine (COM) at the University of Malawi and the University of North Carolina (UNC) Project to build evidence regarding appropriate duration of treatment with amoxicillin dispersible tablets (DT) for chest-indrawing childhood pneumonia in malaria-endemic settings in Africa. An expanded evidence base will contribute to future iterations of integrated community case management guidelines, which in turn will test innovative approaches to childhood pneumonia treatment.

**Type of research:** The proposed approach involves conducting a double-blinded, randomized, non-inferiority trial with the objective to assess the effectiveness of shorter duration amoxicillin DT treatment of chest-indrawing childhood pneumonia in a malaria-endemic region of Malawi.

**Objectives:** The primary objective of this study is to determine whether 3 days of treatment with oral amoxicillin DT in HIV-negative children 2 to 59 months of age with chest-indrawing pneumonia is as effective as 5 days of treatment.

**Methodology:** The study will enroll 2,000 children presenting to Kamuzu Central Hospital or Bwaila District Hospital in Lilongwe, Malawi. Each child will be randomized to either 3 days of amoxicillin DT followed by 2 days of placebo DT or 5 days of amoxicillin DT. Children in the study will be hospitalized for 48 hours after enrollment and will have scheduled study visits at days 2, 4, 6 and 14.

**Expected findings and their dissemination:** We predict that the rates of treatment failure will be similar in both arms and that 3 days of treatment will be non-inferior to 5 days of amoxicillin DT for chest-indrawing pneumonia. Findings from this study will be disseminated through a peer-reviewed journal and shared with the scientific community.

## PROTOCOL OUTLINE

| | **Title:** | **Innovative Treatments in Pneumonia (ITIP) 2**: Double-blind randomized controlled clinical trial of 3 days versus 5 days amoxicillin DT for chest-indrawing childhood pneumonia among children 2-59 months of age presenting to Kamuzu Central Hospital in Lilongwe, Malawi | | --- | --- | | **Sponsor:** | Save the Children Federation, Inc. (SC) | | **Collaborating Organizations:** | Save the Children International – Malawi Country Office  University of Washington (UW)  Malawi Ministry of Health (MOH)  College of Medicine (COM) at the University of Malawi  University of North Carolina (UNC) Project, Lilongwe Medical Relief Fund Trust  Kamuzu Central Hospital (KCH)  Bwaila District Hospital (BDH) | | **Funding Source:**  **Study Products:** | Bill and Melinda Gates Foundation (BMGF)  3 days of 250 mg amoxicillin DT followed by 2 days of placebo DT (intervention) vs 5 days (control) of 250 mg amoxicillin DT in two divided doses based on age bands (500 mg/day for children 2 months up to 12 months, 1000 mg/day for children 12 months up to 3 years, and 1,500 mg/day for children 3 years up to 5 years of age) | | Rationale: | Build evidence regarding appropriate duration of treatment with amoxicillin DT for chest-indrawing childhood pneumonia in a malaria-endemic setting in Africa | | **Population:** | 2,000 HIV-1 seronegative children, ages 2-59 months of age with chest-indrawing pneumonia | | **Schema:** | Eligible volunteers will be randomized in a double-blinded manner in a 1:1 ratio as follows:  ITIP2: chest-indrawing   | Study groups | N | Day 1 | Day 2 | Day 4 | Day 6 | Day 14 | | --- | --- | --- | --- | --- | --- | --- | | 3 days amoxicillin DT  + 2 days placebo DT | 1,000 | X | X | X | X | X | | 5 days amoxicillin DT | 1,000 | X | X | X | X | X | | | **Objectives:** | 1. Primary: treatment effectiveness 2. Secondary: clinical relapse, treatment failure or clinical relapse, adverse events (AEs) and serious adverse events (SAEs); cofactors of malaria, wheeze, oxygen saturation level <93%, moderate malnutrition, very fast-breathing, and age | | **Endpoints:** | 1. Primary:    - Proportion of children failing treatment 2. Secondary:    - Proportion of children with clinical relapse    - Proportion of children with clinical relapse or treatment failure    - Proportion of children with AEs and SAEs    - Proportion of children failing treatment among those testing positive for malaria (overall)    - Proportion of children failing treatment among those with wheeze    - Proportion of children failing treatment among those with oxygen saturation <93%    - Proportion of children failing treatment among those with mid-upper arm circumference (MUAC)-defined moderate malnutrition (11.5-13.5cm)    - Proportion of children failing treatment among those with very fast-breathing for age (>70 breaths per minute for 2–11 months, >60 breaths per minute for 12–59 months)    - Proportion of children failing treatment by age | | **Timeline:** | Projected duration of enrollment is about 30 months.  All children will be followed for 14 days after randomization. | |
| --- | --- | --- | --- | --- | --- | --- | --- | --- | --- | --- | --- | --- | --- | --- | --- | --- | --- | --- | --- | --- | --- | --- | --- | --- | --- | --- | --- | --- | --- | --- | --- | --- | --- | --- | --- | --- | --- | --- | --- | --- | --- |

**Innovative Treatments in Pneumonia 2**

# BACKGROUND AND INTRODUCTION

The burden of childhood pneumonia remains high, and improved access to effective treatment saves lives. Because vaccines cannot prevent all episodes of pneumonia and because pneumonia incidence remains unacceptably high, there is an urgent need to focus on effective and affordable treatment. Treatment of bacterial pneumonia requires an effective antibiotic used in adequate doses for an appropriate duration. The recommended duration of treatment currently ranges between 5 and 14 days. Shorter duration of therapy, if found to be sufficiently effective, could be particularly important in resource-limited settings where there is a high risk of death, poor access to medicines and health care, and limited budgets for medicines. A shorter antibiotic course is much simpler to administer and is more likely to have improved patient adherence compared to a longer course of treatment when delivered in a community setting. Using a study in Zambia of fast-breathing pneumonia as an example of pneumonia treatment in an outpatient setting, researchers identified only 54% adherence to all doses for 5 days of treatment while observing 76% adherence for 3 days of treatment.^[1]^ Improved patient adherence could also prevent the emergence of antibiotic resistance as well as reduce the rates of adverse events associated with antibiotics. Most importantly, it would reduce the burden on the health systems in resource-limited settings by driving down the costs associated with treatment of childhood pneumonia.

Amoxicillin is recommended as the first-line treatment for fast-breathing and chest-indrawing childhood pneumonia.^[2][3]^ For the treatment of chest-indrawing childhood pneumonia, the World Health Organization (WHO) has revised pneumonia case management guidelines, including a new classification of pneumonia severity and a change in first-line antibiotic therapy. Evidence has emerged that has prompted the development of these revised guidelines. In addition to showing that amoxicillin is superior to cotrimoxazole for the treatment of fast-breathing pneumonia, the data show that oral amoxicillin is equivalent to injectable penicillin for the treatment of chest-indrawing pneumonia. A multicenter, randomized, open-label trial conducted by the WHO Acute Paediatric Pneumonia I Study Group at nine tertiary health facilities in eight developing countries compared the efficacy of a 5-day course of oral amoxicillin versus injectable penicillin in 1,702 children aged 3 to 59 months with chest-indrawing pneumonia.^[4]^ The study found treatment failure rates of 19% at 48 hours in each treatment group (n=161 penicillin; n=167 amoxicillin; risk difference -0.4%; 95% CI 4.2–3.3). In England, the PIVOT trial compared a 7-day course of oral amoxicillin versus intravenous benzyl penicillin for the treatment of severe pneumonia and found that among children with radiologically confirmed pneumonia, the two treatments were equivalent, each having a median time of 1.3 days to achieve the primary outcome of temperature below 38°C for 24 continuous hours.^[5]^ In a multicenter, randomized, open-label equivalency trial, the NO-SHOTS study in Pakistan compared high-dose oral amoxicillin (80-90 mg/kg/day) in a twice-daily regimen over 5 days versus 2 days of injectable ampicillin followed by 3 days of high-dose oral amoxicillin in children with chest-indrawing pneumonia; findings showed that amoxicillin given orally twice a day was equivalent.^[6]^ At day 6, there were 87 (8.6%) treatment failures in the hospitalized group and 77 (7.5%) treatment failures in the home-treatment (oral amoxicillin) group (risk difference 1.1%; 95% CI -1.3–3.5).

In two cluster-randomized community-based trials in Pakistan, oral amoxicillin administered in the community was found to be as effective as standard treatment with a first dose of cotrimoxazole and referral to a facility.^[7]^ In these two studies, 7,621 children aged 2 to 59 months with chest-indrawing pneumonia were either given oral amoxicillin (80-90 mg/kg/day in two divided doses) for 5 days (intervention) or the standard treatment of care at the time - a first dose of oral cotrimoxazole followed by referral to the nearest health facility (control). Treatment failure rates by day 6 were significantly reduced in the intervention clusters compared to control clusters (9% versus 18%; risk difference 8.9%) in Haripur District.

Based on the evidence above, the WHO found that in a programmatic context, the distinction between previously defined “non-severe pneumonia” and “severe pneumonia” lost its significance. The revised classification now includes only two categories of pneumonia: 1) “pneumonia” with fast-breathing and/or chest-indrawing, which requires home therapy with oral amoxicillin; and 2) “severe pneumonia,” pneumonia with any general danger sign, which requires referral and injectable therapy.^[8]^ This approach is intended to greatly simplify the management of childhood pneumonia, which will result in a substantially lower need for referral and better treatment outcomes. In the new guidelines, one important distinction between the treatment of fast-breathing and chest-indrawing pneumonia is the duration of oral amoxicillin administration: 3 days for fast-breathing pneumonia cases in areas with low HIV prevalence and 5 days for cases of chest-indrawing pneumonia. With the recent release of the revised WHO guidelines, there is a need to see if a 3-day course of amoxicillin given twice a day is as effective as a 5-day course in the treatment of chest-indrawing pneumonia.

# RATIONALE

There is a critical need for African-specific data, as countries in Africa, including Malawi, endeavor to put into place evidence-based policies and treatment guidelines informed by the local context. There is also a need for local studies in malaria-endemic settings in Africa using child-friendly amoxicillin DT, which the WHO has established as the optimal product formulation for first-line treatment of pneumonia in children less than 5 years of age. ^[18] [19] [20]^ This innovation in first-line antibiotic treatment—being flexible for dose adjustment while remaining within the effective therapeutic range—represents major life-, resource-, and cost-savings to families, health facilities, communities, and local governments. Oral amoxicillin DT is safe and well tolerated. Common side effects typically include rashes, nausea, vomiting, and diarrhea. Oral amoxicillin DT can be easily administered by caregivers in the home, making treatment simpler. However, many settings have been slow to adopt the WHO recommendations on oral amoxicillin as an at-home treatment for pneumonia. The clinical trial described in this protocol intends to provide evidence that could improve access to care by improving case management of pneumonia at the household level, which will lead to improved drug adherence and significantly reduce childhood pneumonia deaths.

# STUDY HYPOTHESIS, OBJECTIVES AND ENDPOINTS

## Study Hypothesis

3 days is non-inferior to 5 days of amoxicillin DT treatment for chest-indrawing pneumonia.

## Study Objectives

The broad objective of this study is to provide scientific evidence assessing the optimal duration of treatment with amoxicillin DT for chest-indrawing childhood pneumonia in the malaria-endemic setting in Malawi, Africa.

- - Primary Objective
    - To determine whether 3 days of treatment with oral amoxicillin DT in HIV-negative children 2 to 59 months of age with chest-indrawing pneumonia is as effective as 5 days of treatment.
  - Secondary Objectives
    - To determine whether the intervention arm has equivalent rates of treatment relapse as the control arm among those without treatment failure before or on day 6.
    - To determine whether the intervention arm has equivalent rates of combined treatment failure and relapse before or on day 14 as the control arm.
    - To determine whether the intervention arm has equivalent rates of adverse events (AEs) and serious adverse events (SAEs) as the control arm.
    - To investigate whether there may be a differential treatment response in children who test positive for malaria at baseline. This information will be useful to plan further childhood pneumonia and malaria integrated interventions in similar settings.
    - To determine whether there is a differential treatment response in enrolled children with wheeze during screening (identified prior to any bronchodilator administration).
    - To determine whether there is a differential treatment response in enrolled children with an oxygen saturation level <93% by pulse oximetry at baseline.
    - To determine whether there is a differential treatment response in enrolled children with mid-upper arm circumference (MUAC)-defined moderate malnutrition at baseline (11.5-13.5cm).
    - To determine whether there is a differential treatment response in enrolled children with very fast-breathing for age at baseline (>70 breaths per minute for 2–11 months, >60 breaths per minute for 12–59 months).
    - To determine whether there is a differential treatment response by age.

## Study Endpoints

- - Primary Endpoints
    - Proportion of children failing treatment, defined as the development of any of the following criteria during the specified time periods:

Any time before or on day 6:

- - - - WHO Integrated Management of Childhood Illness (IMCI) danger signs
      - Severe respiratory distress (e.g., grunting, nasal flaring, head nodding, or severe chest-indrawing).
      - Oxygen saturation < 90% by pulse oximetry
      - Vomiting within 30 minutes of 3 or more scheduled (i.e., not repeat) dose administrations of study product
      - Change in antibiotics prescribed by a study clinician (e.g., switch to a second-line antibiotic or prescription for onset of a co-infection)
      - Death

At or after initial hospitalization discharge assessment (between 42 and 60 hours post-enrollment):

- - - - Documented axillary temperature > 38 ºC with chest-indrawing

At day 6 outcome assessment:

- - - - Documented axillary temperature > 38 ºC
      - Chest-indrawing
  - Secondary Endpoints
    - Proportion of children with clinical relapse between treatment failure assessment and day 14 follow-up visit among all children without treatment failure before or on day 6.
    - Proportion of children with either treatment failure or clinical relapse before or on day 14 (among all randomized children).
    - Proportion of children with AEs and SAEs.
    - Proportion of children with treatment failure among those testing positive for malaria by rapid diagnostic testing (mRDT) at baseline (overall).
    - Proportion of enrolled children failing treatment among those with wheeze during screening (identified prior to administration of bronchodilators).
    - Proportion of enrolled children failing treatment among those with oxygen saturation <93% by pulse oximetry at baseline.
    - Proportion of children failing treatment among those with MUAC-defined moderate malnutrition (11.5-13.5cm).
    - Proportion of children failing treatment among those with very fast-breathing for age (>70 breaths per minute for 2–11 months, >60 breaths per minute for 12–59 months).
    - Proportion of children failing treatment by age at baseline.

# METHODOLOGY

## STUDY DESIGN

This project involves a double-blinded, randomized, non-inferiority trial in children 2-59 months of age from a malaria-endemic setting in Malawi comparing the effectiveness of 3-day to 5-day amoxicillin DT treatment for chest-indrawing, community-acquired pneumonia (ITIP2).

We plan to evaluate treatment with 3 days versus 5 days of oral amoxicillin DT in two divided doses based on age bands (500 mg/day for children 2 months up to 12 months, 1000 mg/day for children 12 months up to 3 years, and 1,500 mg/day for children 3 years up to 5 years of age) among 2,000 children presenting with chest-indrawing pneumonia in a malaria-endemic region of Malawi. Children in the 3-day arm will also receive 2 days of placebo following 3 days of active drug to complete a 5-day course to maintain the blind as to their randomization allocation.

## STUDY SITE

The Save the Children (SC) PI; the local co-PIs, from the College of Medicine (COM) at the University of Malawi and the University of North Carolina (UNC) Project, Lilongwe Medical Relief Fund Trust; and the team of co-investigators will conduct the research at Kamuzu Central Hospital (KCH) in Lilongwe. A 750-bed government facility, KCH is the primary referral hospital for the central region of Malawi, serving a population of approximately 5 million. Up to 30 or 40 children 5 years of age or younger with fast-breathing pneumonia are seen each day in the outpatient department (OPD) at KCH during the peak pneumonia season. The KCH pediatric department alone admits around 22,000 children per year. Over 100 children 5 years or age or younger with chest-indrawing pneumonia are admitted on average each month at KCH, with as many as 260 admissions a month during peak pneumonia season.

One of the major medical training institutions in Malawi, KCH has four full-time on-call pediatricians, two part-time pediatricians, three medical officers, three to six medical interns, 12 full-time clinical officers, and 45 nurses on staff. In the OPD, two to three clinicians with the support of two to three health surveillance assistants manage the triage area. In addition to the triage area, there are also emergency/resuscitation, priority, and low-risk areas in the OPD. The hospital has a high-functioning laboratory unit and a radiology unit that is capable of conducting x-rays (including a mobile unit, which is housed in the pediatric ward), ultrasounds, and computed tomography scans.

Bwaila District Hospital (BDH) is the district hospital for Lilongwe and is located in the central business district of Lilongwe. Approximately 200 to 250 children present to the BDH OPD per day. There are no inpatient facilities for children at BDH, and those requiring inpatient care are referred to KCH.

## STUDY POPULATION

## Study Population Overview

Although KCH draws from a large catchment area in the central region of Malawi, children eligible for this study are to be from the Lilongwe District. Malawi is ranked 174^th^ in the United Nations Development Programme’s human development index, and almost 89% of the working population earns less than $2USD a day.^[9]^ Lilongwe includes large peri-urban settlements with crowded living conditions and without adequate sanitation infrastructure. Overall, the nation’s adult literacy rate is 61%. Life expectancy at birth is 55.31 years and the under-five mortality rate is 71 deaths per 1000 children.

Malaria is endemic in Malawi with the highest prevalence of malaria parasitaemia in children between 6 and 36 months of age (60.1%).^[10]^ The incidence of malaria in the area is highest during the rainy season, between December and April each year. Malawi’s adult HIV prevalence was estimated to be 10.3% in 2013. There were 170,000 estimated HIV-positive children 14 and younger. HIV is more prevalent in urban communities than rural areas.^[11]^

We expect study participants to be representative of the ethnic demographics in the area. We anticipate enrolling equal numbers of female and male children for a total participant population of 2,000 volunteers for this trial.

## Participant Eligibility

Study participants will be HIV-1 seronegative children 2-59 months of age who present to KCH or BDH with chest-indrawing. Volunteer families will be recruited and screened, those whose children are determined to be eligible, based on the inclusion / exclusion criteria, will be enrolled in the study and followed for 14 days. Recruitment, screening and enrollment can occur at KCH or BDH. Hospital observation or admission, and follow-up will occur at KCH. Final eligibility determination will depend on the results of the medical history, clinical examination, appropriate understanding of the study and completion of the consent process.

Case definition of chest-indrawing pneumonia:

- - Cough <14 days or difficulty breathing
  - Visible indrawing of the chest wall, with or without fast-breathing

## Inclusion Criteria

- - Male or female, 2 to 59 months of age.
  - History of cough <14 days or difficult breathing with chest-indrawing.
  - Ability and willingness of child’s caregiver to provide informed consent and to be available for follow-up for the planned duration of the study, including accepting a home visit if he/she fails to return to KCH for a scheduled study follow-up visit.

## Exclusion Criteria

- If chest-indrawing observed at screening resolves after bronchodilator challenge, among those with wheeze at screening.
- Severe respiratory distress (e.g., grunting, nasal flaring, head nodding, or severe chest-indrawing).
- Presence of WHO IMCI danger signs including: lethargy or unconsciousness, convulsions, vomiting everything, or inability to drink or breastfeed.
- Hypoxia (SaO_2_ < 90% on room air, as assessed by a Lifebox pulse oximeter).
- Stridor when calm.
- HIV-1 seropositivity or HIV-1 exposure, assessed as follows:
  - An HIV-positive result upon rapid antibody test will exclude any child from this study.
  - If a child is less than 12 months or age with a positive rapid test result, the child will be referred to receive additional confirmatory HIV testing (e.g., dried blood spot filter paper test) and follow-up from KCH staff, as per standard of care. Even if the confirmatory HIV testing subsequently shows that child is HIV-negative, he or she will remain excluded from the study.
  - If a child is less than 24 months of age and has an HIV-negative result upon rapid antibody test, the child’s biological mother’s HIV status will need to be assessed. If the mother is HIV-positive, the child will be excluded. If the mother has a documented HIV-negative test result from within the past 6 weeks, the child will be included. If the mother does not have documentation of an HIV-negative test result, she will be tested via rapid antibody testing to determine the child’s eligibility for this study.
  - If a child is over 24 months of age, an HIV-negative rapid antibody test is required for inclusion in the study.
  - Note: If a child has documentation of an HIV-negative test result from within the past 6 weeks, that test result will be used for the child’s eligibility assessment according to the algorithm described above.
- Severe acute malnutrition (i.e., weight for height/length < -3 SD, mid-upper arm circumference <115 mm, or edema).
- Possible tuberculosis (coughing for more than 14 days).
- Severe anemia, classified by WHO pocketbook guidelines (i.e., severe palmar pallor or hemoglobin <8.0 g/dL)
- Severe malaria, classified by WHO pocketbook guidelines (e.g., positive mRDT with any danger sign, stiff neck, abnormal bleeding, clinical jaundice, or hemoglobinuria)
- Known allergy to penicillin or amoxicillin.
- Receipt of an antibiotic treatment in the 48 hours prior to the study based on caregiver’s self-report and/or documentation in child’s medical record.
- Hospitalized within 14 days prior to the study.
- Living outside Lilongwe urban area, the study catchment area.
- Any medical or psychosocial condition or circumstance that, in the opinion of the investigators, would interfere with the conduct of the study or for which study participation might jeopardize the child’s health.
- Any non-pneumonia acute medical illness which requires antibiotic treatment per local standard of care.
- Participation in a clinical study of another investigational product within 12 weeks prior to randomization or planning to begin participation during this study.
- Prior participation in an Innovative Treatments in Pneumonia study during a previous pneumonia diagnosis.

## STUDY PERIOD

Each child will be followed for 14 days after enrollment. Projected duration of enrollment is anticipated to be about 30 months for this study. The high volume of children presenting to the OPD each day with pneumonia is expected to exceed the capacity of study staff to adequately assess each child for this study, so a maximum of 15 children for this trial will be enrolled each day. To avoid potential selection bias, each day children will be screened for enrollment in a sequential manner, as much as possible. Children that are not assessed for this study will receive the standard of care at KCH or BDH, which includes 5 days of oral amoxicillin, hospitalization, and treatment of any co-infection.

The funding for this study is for three years. This period includes the time required to prepare the necessary documents for the study, train all study personnel, initiate the study site, conduct the study and all data collection procedures, clean and analyze the data, and prepare the results for publication and presentation.

## SAMPLE SIZE

Refer to Section 5, Statistical Design and Analysis, for more details.

A total of 2,000 children (1,000 per treatment group) will be enrolled. With this sample size we aim to rule out a relative increase in failure rate of 50%. The power of the study to show non-inferiority of the 3-day treatment group at the final analysis when both treatment groups have exactly the same failure rate depends on the failure rate in the standard treatment (5-day) group. The estimated power is adjusted for a 5% loss to follow-up rate in each of the arms. For example, with 1,900 children (950 per treatment arm) for the primary analysis this study has 88% power to show non-inferiority of the 3-day treatment group if the failure rate in both groups is 8% and the non-inferiority margin is a 50% relative increase (which for this failure rate is equivalent to an absolute non-inferiority margin of 4.0%). For this failure rate in the standard treatment group (of 8%), at the final analysis the 95% confidence interval would exclude 12.0%, even if the failure rate is as large as 9.7% in the 3-day treatment group. With 1,900 children with complete outcome data in the primary analysis, the power of this study to show non-inferiority ranges from 64.8% if the failure rates in both groups are 4%, to 96.5% if the failure rates in both groups are 12% (assuming a relative non-inferiority margin of 50% (or equivalently a factor of 1.5). Further details will be provided in the Statistical Analysis Plan (SAP).

## STUDY PROCEDURES

Refer to Appendix I for Study Procedures and Visits Table. Refer to Appendix II for Laboratory Specimens Collection, Timing and Distribution Table.

Note that at study initiation, a pilot study will be conducted with up to 100 participants. For these participants, all study procedures as outlined below will be followed EXCEPT for the study product allocation. Pilot participants will undergo mock randomization as all participants in the pilot phase will receive 5 days of active drug. The purpose of this pilot study is to ensure study feasibility, safety and conduct prior to allocating study product based on randomization. Participants in the study pilot will not be informed of the mock randomization process or that they all received active drug. Concealing this will allow study staff to evaluate procedures and participant compliance in a situation that exactly mirrors that of the clinical trial. All data collected on participants enrolled in the pilot study will be entered into a separate database and will not be analyzed with the data from the main trial. The target enrollment of 2,000 participants for the main trial does not include those participants enrolled in the study pilot.

**Figure 1.** Study Flow Diagram

Recruitment

Screening

Enrollment and Randomization

(Day 1 Visit)

*Hospitalization for all children for 2 days*

Intervention arm

(3 days amoxicillin)

Day 2 Visit

Day 4 Visit

Day 6 Visit

Day 14 Visit

(Study exit)

Control arm

(5 days amoxicillin)

Day 2 Visit

Day 4 Visit

Day 6 Visit

Day 14 Visit

(Study exit)

## Recruitment

Recruitment for this study will be performed by KCH staff during routine intake and screening procedures for the OPD at KCH or BDH. Children between 2 to 59 months of age presenting to the OPDs with cough or difficult breathing will be assessed by hospital staff for potential referral to the study. For any children with a cough fewer than 14 days and chest-indrawing, the clinician will read to the caregiver an ITIP recruitment script (refer to Appendix IV) with a brief introduction to the study. If the caregiver is interested in learning more about the study and in potentially having the child assessed for eligibility, he/she will be referred to study staff.

All KCH staff involved in recruitment procedures will be trained in relevant study-specific procedures and certified in GCP. Each recruitment and referral interaction will be documented for study records.

## Screening

Screening procedures are conducted by study staff to determine eligibility for enrollment in the study. All inclusion/exclusion criteria must be assessed on presentation. The following procedures are performed for screening:

- - Provide information on the study
  - Obtain written informed consent for screening
  - Assign participant identification (ID) number
  - Collect demographic and address information
  - Collect medical history
  - Assess all eligibility criteria, including respiratory rate, chest-indrawing and pulse oximetry assessments (if not already documented in the medical record from that day) as well as a targeted physical examination
  - Perform malaria rapid diagnostic testing (mRDT). Those who are found to have malaria will receive appropriate antimalarial treatment using artemisinin-based combination therapy in addition to the randomly assigned treatment for pneumonia
  - Perform HIV rapid antibody testing if HIV status unknown
  - Perform hemoglobin test (HemoCue®) for anemia

Note that if a child presents with wheezing (audible or auscultatory), study staff will administer a trial of rapid acting inhaled bronchodilator for up to three times, 15-20 minutes apart. Study staff will then assess for fast-breathing and chest-indrawing again to determine the child’s eligibility for this study.

All screening procedures will be conducted by study staff, with the possible exception of the HIV rapid antibody test. HIV testing may be performed by either study staff or a team at KCH or BDH specially trained and experienced in pediatric HIV counseling and testing, whichever will reduce wait times for potential study participants and minimize disruption in regular care provision at KCH or BDH. Caregivers will be informed of all screening results during the screening visit, regardless of the eligibility status of their child.

For those children who are not eligible, study staff will inform the caregiver(s) that their child will not be able to participate in the study and will receive standard care at KCH or BDH instead. Children less than 12 months of age or breastfeeding who are excluded based on an HIV-positive rapid antibody test result will be referred for confirmatory testing (e.g., dried blood spot filter paper test).

All screening procedures will be documented in the appropriate study forms, including logs and case report forms. Clinical assessments and findings will also be documented in the child’s medical record, as appropriate.

## Informed Consent

For the purposes of this protocol, “caregiver” refers to the legally authorized representative (LAR) of the child and informed consent may only be obtained from a child’s LAR. Both mother and father are considered LARs for a child, so consent may be obtained from either parent. In the absence of a biological parent, documented proof of legal guardianship would be needed to establish a caregiver’s status as a LAR.

This study will have two informed consent forms (ICFs): one for screening procedures and one for enrollment procedures. Informed consent is the process of ensuring that caregivers of children fully understand what will and may happen to their children while participating in a research study. Study staff will administer a comprehension checklist to potential participants’ caregivers prior to obtaining written informed consent to ensure that caregivers fully comprehend the nature of the study. The informed consent process continues throughout the study. Key study concepts will be reviewed periodically with the caregivers and the review will be documented. Additionally, if any new information is learned that may affect the caregiver’s decision to stay in the trial this information will be shared with the caregivers in writing. All consent materials will be approved by the appropriate Institutional Review Board (IRB) and Independent Ethical Committee (IEC) prior to use.

Refer to detailed description of informed consent procedures and ethical committee approval in Section 6 (Ethical Considerations and Consent).

## Enrollment Visit

After screening is complete, study staff will perform the enrollment visit procedures for the trial for only those children who are still eligible. For those children who are eligible, the following procedures are performed for enrollment:

- - Administer comprehension checklist
  - Obtain written informed consent for enrollment
  - Perform a physical exam including vital signs and an assessment of any baseline characteristics not already recorded in the medical record or assessed during screening, including measurement of MUAC
  - Collect vaccination history and additional socio-demographic information
  - Collect locator information to be able to contact caregiver and conduct a home visit, if necessary
  - Follow procedures for randomization assignment
  - Provide the participant caregiver with the appropriate study product kits (described below).
    - A study nurse or clinician will prepare and administer the first drug dose, carefully instructing the caregiver how to administer subsequent doses appropriately. The caregiver will receive an instruction sheet with visual and text descriptions of the timing and dosing necessary to complete the treatment regimen. The pharmacists will be unblinded to the randomization allocation, but both provider and caregiver will be blinded to what the child will receive. The amoxicillin DT and placebo DT will appear and taste the same.
    - Children with chest-indrawing will receive amoxicillin DT in two divided doses based on age bands (500 mg/day for children 2 months up to 12 months, 1000 mg/day for children 12 months up to 3 years, and 1,500 mg/day for children 3 years up to 5 years of age) for 5 days (control) or amoxicillin DT in two divided doses based on age bands for the first 3 days followed by placebo DT in two doses for 2 days (intervention).

All enrollment procedures will be documented in the appropriate study forms. Clinical assessments and findings will also be documented in the child’s medical record, as appropriate.

## Randomization

Randomization and enrollment occur at the same study visit, designated Day 1. Randomization is defined as the process of assigning a child to a study arm; assignments are computer-generated by the Protocol Statisticians at UW. The study pharmacists on site will receive the randomization list from the Protocol Statisticians and will be responsible for recording the blinded portion of the randomization code on each study participant’s case report forms and blister pack when they are enrolled and randomized. The list that contains a link between the allocation arm and the study participant ID will be maintained by the study pharmacists under lock and key and/or electronic encryption. Other study staff will not have access to the randomization list.

Children will be randomized to 3 days of oral amoxicillin DT followed by 2 days of placebo DT (intervention) or 5 days of oral amoxicillin DT (control). Treatments will be allocated in a 1:1 ratio. Study investigators and staff will be blinded to all elements of the randomization allocation for the duration of the study.

## Management of Study Participants During Hospitalization

All children enrolled in this chest-indrawing study (ITIP2) will be initially admitted to the hospital for 2 days, to be assessed by study staff for discharge on the morning of Day 3, between 42 and 60 hours after admission. Discharge criteria will be met if none of the criteria for treatment failure are present. Note that due to logistical or social factors (e.g., caregiver not present, lack of transport options at that time of day), some children who meet discharge criteria might not leave the hospital until after the 60 hour window. When confirmed by a study investigator, such children will not qualify as failing treatment, so long as they do not meet any of the other treatment failure criteria.

Children will be primarily managed by study clinicians during any hospitalization, including ward rounds and clinical assessments. Diagnostic tests and medication for intercurrent illnesses will be ordered per ward protocols with results documented in study files. This includes antibiotic treatment regimen changes. To ensure adherence to study protocol, study staff will administer study drugs to participating children during any hospitalization. Study clinicians will be informed by hospital clinicians about the clinical care of children in the study and any clinical decisions made by hospital staff. Study staff will be responsible for orders in the event that study-related laboratory tests and specimen collection are required. In addition to all study staff, all KCH clinicians and nurses will undergo GCP training.

## Follow-Up Visits

Target dates for follow-up visits are calculated from Day 1, the date of randomization. All visits must occur on the calendar day on which they are initially scheduled or within 24 hours afterwards, with the exception for the Day 14 visit, which can occur either 2 days before or after day 14 and still be considered completed within the visit window.

Children will be in the hospital for a follow-up visit on day 2. Caregivers will bring their children for follow-up visits on days 4, 6, and 14.

The Day 2 study visit will occur while the child is still hospitalized for all children admitted for 2 days in ITIP2. Prior to discharge, caregivers will be instructed on how and when to administer the study drug to their child as well as how to contact the study site personnel for concerns that may arise between scheduled visits. Caregivers will receive an instruction sheet with details on the timing and dosing necessary to complete the treatment regimen as well as signs and symptoms that should prompt an immediate call to study staff. A study phone number will be provided to each caregiver and will be answered 24/7.

If a child is not still in the hospital, study staff will attempt to contact the caregiver by phone prior to scheduled study visits to remind them to return to the clinic at the appropriate time.

Follow-up visit procedures at scheduled visits include the following:

- - Review/update locator information
  - Review results from prior visits
  - Collect medical history since the last study visit
  - Perform physical exam including respiratory rate, chest-indrawing and pulse oximetry assessments to assess for treatment failure or clinical relapse
  - For all visits EXCEPT Day 14 visit:
    - Collect study product adherence information from caregiver
    - Review drug dosing and administration procedures with caregiver
  - At the outcome assessment visit (Day 6):
    - Conduct pill counts of study product and document unused amounts
    - Collect all unused study product from the caregiver

For adherence, note that completing 80% of all scheduled dose administrations is considered to meet treatment completion criteria (i.e., 8 out of 10 doses over the 5 days). If doses are missed due to non-adherence, no study action will be taken beyond documenting the missed doses and counseling the caregiver on adherence and study product administration. If a child vomits within 30 minutes of a dose, one repeat dose may be attempted. If a child vomits within 30 minutes after 3 or more scheduled (i.e., not repeat) dose administrations, this will be considered a treatment failure and that child will be referred to care for a work-up of the vomiting cause and will be prescribed a course of second-line antibiotics.

All follow-up visit procedures will be documented in the appropriate study forms. Clinical assessments and findings will also be documented in the child’s medical record, as appropriate.

## Missed Visits

In case of a no-show at the clinic for a scheduled study visit, study personnel will call the caregiver and visit the child’s home either that afternoon or the following day to conduct the study visit. Maximum efforts will be made to ensure complete follow-up in the trial. For children who do not complete a scheduled visit within the visit window, that visit will be documented as “missed” but study staff will still attempt to complete the appropriate assessments from that visit, if possible (e.g., Day 6 visit performed and documented on Day 9).

Children who miss a visit, for other than a protocol-mandated reason for discontinuation, are permitted to continue with any subsequent study treatments that can still be scheduled in the time interval specified by the protocol.

Based on our current experience, we expect that fewer than 5% of the children will be lost to follow-up at the time of primary outcome assessment. We think it is unlikely that attrition rates will differ between randomization groups.

## Interim Contacts and Visits

Interim contacts and visits (those between regularly scheduled follow up visits) may be performed at caregiver request or as deemed necessary by the site investigators or designee at any time during the study. All interim contacts and visits will be documented in the child’s study records and on applicable case report forms (CRF). Interim visits may occur at the study clinic or at the child’s home.

## Therapy for Treatment Failure and Clinical Relapse

If a child is determined to have treatment failure on or before Day 6 or clinical relapse between Day 6 and Day 14, he or she will be hospitalized and will receive second-line therapy. At KCH, standard of care for children failing oral amoxicillin is to receive benzyl penicillin and gentamycin. Enrolled children failing treatment or experiencing clinical relapse will receive this regimen as inpatients, regardless of their randomization allocation.

## Withdrawal and Early Termination

Children and their caregivers may voluntarily withdraw from the study for any reason at any time. The site investigators may also withdraw children from the study in order to protect their safety if, in the investigators’ opinion, continuing participation would jeopardize the child’s health. Any participant withdrawal or early termination will be documented in the appropriate study forms.

If a child withdraws from the study during the first 3 days of treatment, s/he will be referred to care with the recommendation that the child receive oral amoxicillin for a full 5-day treatment course. If a child withdraws from the study after completing the first 3 days of treatment, but before completing the full 5 days of study product, the child will be referred to care with the recommendation that s/he receive an additional 2 days of treatment with oral amoxicillin.

## Study Termination Visit

The Day 14 visit will serve as the study termination visit. Procedures for this visit, in addition to the standard follow-up visit procedures described above, include the following:

- - Collect any unused study product from caregiver, if not retrieved at prior study visit
  - Refer child to clinical care, as needed
  - Document contact in child’s study records

## Biohazard Containment

As exposure to blood-borne pathogens can occur through contact with contaminated needles, blood, and blood products, appropriate blood and secretion precautions will be employed by all personnel in the HIV, anemia, and malaria testing for this study as recommended by the U.S. Centers for Disease Control. Biohazardous waste will be contained according to institutional, transportation/carrier, and all other applicable regulations.

## STUDY PRODUCTS

## Presentation and Formulation

Prepared study product will be labeled by the manufacturer so as to maintain the blind; both the amoxicillin DT packages and the placebo DT packages will have the same printed information (e.g., “ITIP Study Product - dispersible tablets 250 mg”). This is a double-blinded study in which the study drug assignment will be concealed from the child, caregivers, and study personnel (with the exception of the study pharmacists and the Protocol statisticians). Labels will meet all national and local requirements. The label must also include the product expiry date, batch number, manufacture date.

For this study, amoxicillin DT is supplied as round, orange, uncoated tablets that contain 250 mg of amoxicillin and inactive ingredients. The placebo formulation for this study is a dispersible tablet that appears, tastes, smells, and disperses indistinguishably from the amoxicillin DT, although it is composed of only the inactive ingredients.

Study drug (amoxicillin DT and placebo DT) will be supplied in bulk shipments by a Sponsor-contracted drug manufacturer to the study site. Active drug and placebo pills will be sent in separate shipment packaging to avoid any mis-identification on the part of the study pharmacists, the only un-blinded study staff members in Malawi. The study pharmacists will prepare study product kits in batches based on the stratified randomization list. The amoxicillin DT and placebo DT will be individually labeled by the study pharmacist with each child’s participant ID number printed on self-adhesive sticking labels. Different ID sequences and/or label colors will be used for the three age bands to minimize the chances of a prescription error. The link between the participant ID numbers and randomization code will be kept securely by the study pharmacists.

Amoxicillin DT is commercially available in blister packs containing 10 tablets per blister pack. Each child’s study product supply will be re-packaged by the study pharmacists in kits based on the child’s age band and randomization allocation. Each kit will represent the entire study product supply for one child. A supply of 5 days-worth of study product will be dispensed based on the child’s age band. Each child’s kit will consist of a package of amoxicillin tablets labeled for the first 3 days and a separate package labeled for days 4 and 5 of treatment, either placebo or amoxicillin (refer to Table 1 below).

**Table 1.** Study Product Kits of Amoxicillin DT or Placebo DT, to be taken twice daily

| **Age Band** | **No. of tablets provided** | |
| --- | --- | --- |
|  | **Days 1-3*** | **Days 4-5** |
| 2 months up to 12 months | 6 | 4 |
| 12 months up to 3 years | 12 | 8 |
| 3 years up to 5 years | 18 | 12 |

*These tablets will all have active drug, regardless of randomization allocation. All other tablets will be either placebo or active drug, based on randomization arm.

## Preparation and Administration

The amoxicillin DT will be provided in 250 mg doses according to the age bands noted in Table 2 and will be administered orally to the child in divided doses twice daily by dispersing in a small amount of clean water or breast milk and provided.

**Table 2.** Study Product Administration by Age Band

| **Age Band** | **Oral Amoxicillin/Placebo Dispersible Tablets (DT)** | |
| --- | --- | --- |
|  | No. of 250 mg tablets, given two times daily | Total study product administered per day |
| 2 months up to 12 months | 1 | 500 mg |
| 12 months up to 3 years | 2 | 1,000 mg |
| 3 years up to 5 years | 3 | 1,500 mg |

Study drug will be maintained and dispensed to the participant caregiver by a study pharmacist. As noted above, the study pharmacists will package tablets received as bulk study drug. Tablets will be used as supplied, meaning that other than packaging and labeling, there is no further study drug preparation required. Study drug is administered orally in either clean water or breast milk, as appropriate. For breast milk administration, the mother will need to express at least 5-10 ml of breast milk into a clean container before dispersing the study product into the liquid. If a mother is unable or unwilling to express milk at the time of study drug administration, clean water can be used instead. For dispersal of study product in water, at least 5-10 ml of bottled, filtered or boiled water can be used in a clean container. Once placed in the liquid, the tablet should be allowed to completely disperse (after at least one minute) before providing the solution to the child to drink. Flavoring agent may be added to the liquid, if desired.

All children, caregivers and research staff will be blinded as to whether the child is in the amoxicillin DT or placebo DT treatment group until the end of the study once the decision to break the study blind is determined by the Sponsor (after completion of study primary manuscript). Codes linking randomization number for each child to actual treatment will be secured in a sealed, opaque envelope and maintained in a locked drawer in the research pharmacy.

Caregivers will be given the emergency contact number for the study personnel during the consenting process in order to report any adverse events.

## Stability and Storage

All study product not already dispensed to children will be stored in locked cabinets only accessible to the study pharmacists, study clinician, and investigators. The study product does not need to be refrigerated and will be stored in a dry location at ambient room temperatures below 25° C (77° F). Study product will be dispensed to participant caregivers as participant-specific kits by the study pharmacist and documented as such.

## Accountability and Disposal

The study pharmacists are required to maintain complete records of all study products received from the Sponsor and/or drug manufacturer and will be responsible for maintaining an accurate record of the randomization codes, inventory, and an accountability record of amoxicillin DT and placebo supplies for this study. The study pharmacists will also be responsible for ensuring the security of these documents, maintaining them under lock and key and/or electronic encryption. Partially used amoxicillin DT and placebo will not be used for human administration.

At the completion of the study, the study pharmacists and site investigators (or designee) will conduct and document a final reconciliation of all study product shipped, received, dispensed, consumed, and remaining. Any discrepancies identified will be investigated, resolved, and documented before any unused study drug is destroyed. After all accounting and reconciliation procedures are complete and approved by the Sponsor, all unused study product will be destroyed on site and documented in the master study files.

## DATA COLLECTION

Clinical research data will be maintained through a combination of secure electronic data management system and physical files with restricted access. Data related to study endpoints will be extracted from the electronic databases for statistical analysis. Three distinct study databases will be created and maintained: the primary study database with study visit data, a safety database with SAE assessments, and a database with participating children’s personally identifiable information. The first two study databases containing study endpoint data will identify children only by study identification numbers and will not contain identifying information such as name, address, medical record number or personal contact information. In the third database, the study coordinator will maintain a log that will contain the link between personal identifiers and the study participant IDs. The linklog and any other documentation (paper-based or electronic) that has both personal identifiers and the participant ID will have restricted access and will be stored in a secure manner separately from other study data and will be retained for at least five years after the last participating child exits the study.

## Case Report Forms

All study data will be collected by the clinical study staff using designated source documents or paper-based case report forms (CRFs). Study data will be entered directly into the CRFs during a study visit. Data from the paper-based CRFs will be entered after the fact into the electronic database as promptly as is feasible. Study staff will maintain source documents for each child at the study site. Source documentation will be available for review to ensure that the collected data are consistent with the CRFs. CRFs and laboratory reports will be reviewed by the site clinical team who are responsible for ensuring that they are accurate and complete. CRFs, source documents and other supporting documents (both electronic and paper-based) will be kept in a secure location and remain separate from participant identification information (name, address, etc.) to ensure confidentiality. Standard Good Clinical Practices (GCP) practices will be followed to ensure accurate, reliable and consistent data collection.

## Source Documents

Source documents include but are not limited to:

- Signed informed consent forms
- Documentation of the comprehension checklist
- Visit documentation that includes dates of study visits
- Receipts for travel reimbursement
- Reported laboratory results
- Clinic notes

A copy of all laboratory results will also be included in the child’s medical records. Site investigators will maintain, and store in a secure manner, all source documents throughout the study. These documents will be retained for at least five years after the last child exits the study.

## DATA MANAGEMENT

Primary data management activities will be undertaken by the designated contract research organization (CRO). The on-site study data manager will oversee data-related procedures at the study site and will be supervised by the CRO data management staff. Data management activities include data entry and validation, data coding and cleaning, database quality control, disaster recovery plans, adverse event reporting and tracking systems, preparation and submission of safety and compliance reports to the sponsor, and preparation of final study database. Data management activities will be performed using Clindex® Clinical Trial and Data Management software, developed by Fortress Medical Systems.

## Data Access

The participating site will maintain appropriate medical and research records for this trial, in compliance with International Conference on Harmonization Good Clinical Practice E6 (ICH-GCP), regulatory, sponsoring organization and institutional requirements for the protection of confidentiality of children. The site will permit authorized representatives of the sponsor and regulatory agencies to examine (and when required by applicable law, to copy) clinical records for the purposes of quality assurance reviews, audits and evaluation of the study safety and progress. User-specific usernames and passwords are required to log onto the database. User rights will be provided to study staff, PIs, and co-investigators at the level appropriate for each individual’s job description.

## Data Storage

The site investigators and designees will maintain, and store securely, complete, accurate and current study records throughout the study. In accordance with regulations, study staff will retain all study records on site for at least five years after study closure. Study records will not be destroyed prior to receiving approval for record destruction from the sponsor. Applicable records include source documents, site registration documents and reports, informed consent forms, and notations of all contacts with the child.

The Clindex® database is hosted by Fortress Medical Systems through their Software as a Service platform and accessed remotely online. All of the servers that host the Clindex® software and data are housed at ATOMICdata, a Tier 3, SOC 3 Certified Data Center. The primary hosting facility is at the ATOMICdata Minneapolis South facility.

## External Study Monitoring

The Study Sponsor and other regulatory authority inspectors or their authorized representatives are responsible for contacting and visiting the study site for the purpose of inspecting the facilities and, upon request, inspecting the various records of the trial. Participant confidentiality will be respected. Site monitoring visits will be conducted to assess compliance with ICH-GCP guidelines. Study monitors will visit the site to:

- Verify compliance with human subjects and other research regulations and guidelines
- Assess adherence to the study protocol and study-specific procedures manual
- Confirm the quality and accuracy of information collected at the study site and entered into the study database
- Assess the resolution of any past or ongoing issues identified at previous monitoring visits

The site investigators will allow study monitors to inspect study facilities and documentation (e.g., informed consent forms, clinic and laboratory records, other source documents, case report forms), as well as observe the performance of study procedures. Medical records containing identifying information may be made available for review when the study is monitored by the sponsor or an authorized regulatory agency. Direct access may include examining, analyzing, verifying, and reproducing any records and reports that are important to the evaluation of the study. Site visit logs will be maintained at the study site to document all visits.

## SAFETY ASSESSMENTS AND REPORTING

## Safety Monitoring

This protocol has extensive safety monitoring in place. The study site investigators will be responsible for close safety monitoring of all children participating in the study, and for alerting the protocol team if unexpected concerns arise. Children with chest-indrawing pneumonia will be hospitalized for the first two days to ensure initial continuous monitoring by hospital staff. Each participating child will be evaluated by a study clinician at each study visit. If a child misses a study visit, home visits will be conducted by trained study staff to ensure clinical evaluation. Every effort will be made to trace all children in the study for the final outcome assessment. An emergency number will be provided to all participants’ caregivers so that an on-call clinician can be reached at any time during study participation. As needed, children in the study may be evaluated at interim visits and/or referred for additional care. SAEs will also be regularly reviewed by the study’s CRO safety monitor and medical expert and compiled into reports for the protocol team. The protocol team may seek independent expert medical opinion as the need arises. In addition, an external group, the Data Safety and Monitoring Board (DSMB) will be closely involved in regular safety monitoring.

## Data Safety and Monitoring Board (DSMB)

An independent DSMB will be set up to regularly (approximately every 6 months) review cumulative safety and study conduct data. At a minimum, safety data presented to the DSMB will include summaries of data on AEs, SAEs, adherence rates, treatment failure, and clinical relapse. The DSMB will include at least one pediatrician, one pneumonia expert, and one biostatistician. As the interim analyses are available, the DSMB will review interim comparisons of the trial arms after enrollment has begun. The content, format and frequency of safety data reports will be agreed upon by the protocol team and the DSMB in advance of study implementation, to be documented in a DSMB charter. The DSMB may review the unblinded treatment regimens of individuals, if warranted. The DSMB reviews will be summarized with recommendations to the study Sponsor, as to whether or not there are safety concerns and if the study should continue without change, be modified, or terminated.

In the unlikely event that the protocol team has serious safety concerns that lead to a decision to discontinue study product allocation for all children in the study and stop accrual into the study, the protocol team will request an emergency review of the data by the DSMB before recommending that the study be permanently stopped. At the protocol team’s request, accrual into the study may be temporarily halted before the DSMB has the opportunity to review the relevant data by treatment arm.

## Adverse Events

Per ICH GCP guidelines, an adverse event (AE) is “any untoward medical occurrence in a patient or clinical investigation subject administered a pharmaceutical product and which does not necessarily have a causal relationship with this treatment. An adverse event (AE) can therefore be any unfavorable and unintended sign (including an abnormal laboratory finding), symptom, or disease temporally associated with the use of a medicinal product, whether or not related to the medicinal product.” These come to the attention of site clinicians through interim medical histories, physical examinations and laboratory testing. Study participants’ caregivers will be instructed to contact the study site staff to report any AEs they may experience. In the case of a life-threatening event, they will be instructed to seek immediate emergency care. Where feasible and medically appropriate, participant caregivers will be encouraged to seek medical care for their children where the study clinician is based, and to request that the clinician be contacted upon their arrival.

All AEs will be managed by the clinical study site team in accordance with good medical practices and the standard clinical practices in place at the hospital. The clinical team will assess and treat or refer the participating child for medical care as appropriate, which may include additional study visits, if necessary. If any acute treatment or medical care is required as a result of harm caused by a study product or study procedure, this care will be provided by the site free of charge. All children in the study with an AE will be followed clinically until the AE resolves (returns to baseline) or stabilizes.

## Serious Adverse Event

Serious adverse events (SAEs) will be defined per US 21Code of Federal Regulations (CFR) 312.32 guidelines, or the equivalent Malawi regulations, as AEs occurring that:

- - Result in death
  - Are life-threatening adverse events
  - Require inpatient hospitalization or prolongation of existing hospitalization
  - Result in persistent or significant disability/incapacity, or
  - Are congenital anomalies/birth defects.

Important medical events that may not result in death, be life-threatening, or require hospitalization may be considered serious when, based upon appropriate medical judgment, they may jeopardize the health of the participating child or require medical or surgical intervention to prevent one of the outcomes listed above.

Note that the initial hospitalization for children in ITIP2 does not count as an SAE as the condition for which the child was hospitalized occurred prior to administration of the study product, classifying it as a pre-existing condition and not an AE. If that initial hospitalization is prolonged past the expected duration of 2 days, because the child’s health has deteriorated or the child has not responded to the treatment and still needs hospital supervision, it will be reported as an SAE. Any readmission to the hospital will also be reported as an SAE. Please refer to Section 4.6 on Study Procedures, specifically the portion titled “Management of Study Participants During Hospitalization,” for further detail on discharge assessments and prolonged hospitalization.

All treatment failures will be considered SAEs.

## Adverse Event Relationship to Study Product

The relationship of all AEs to study product will be assessed as follows:

- - Definitely related: adverse event and administration of study agent are related in time, and a direct association can be demonstrated with the study agent.
  - Probably related: adverse event and administration of study agent are reasonably related in time, and the adverse event is more likely explained by the study agent than by other causes.
  - Possibly related: adverse event and administration of study agent are reasonably related in time, and the adverse event can be explained equally well by causes other than the study agent.
  - Probably not related: a potential relationship between administration of study agent and adverse event could exist, but is unlikely, and the adverse event is most likely explained by causes other than the study agent.
  - Not related: the adverse event is clearly explained by another cause unrelated to administration of the study agent. Reportable events must have documentation to support the determination of “not related”.

The assessment for AE relationship to study product must be conducted while the reviewer is blinded to randomization allocation for the child in question.

The initial determination of AE relationship to study product will be made by study staff with as needed consultation with the local PI. A study team medical officer will review determinations of AE relationship and assign the final relationship determination for all Grade 4 and 5 events, including all SAEs. For any death in the study, an independent medical officer will make the final determination of relationship to study product.

## Grading Severity of Events

All adverse events will be graded by the widely used DAIDS AE Grading Table Version 1.0, December 2004; clarification August 2009. This grading table is now adopted by the U.S. Food and Drug Administration (U.S. FDA) for AE reporting. This table is available at: <http://rcc.tech-res.com/Document/safetyandpharmacovigilance/Table_for_Grading_Severity_of_Adult_Pediatric_Adverse_Events.pdf>

## Safety Reporting

All SAEs must be reported by the site to the medical officers and Sponsor within 24 hours. Attribution with regard to relationship to study product will only be reported for AE grades 2 or above and for all SAEs. Prior to study unblinding, any Grade 4 local or systemic reactogenicity symptom or AE, described by the site staff as possibly, probably, or definitely related to the study product, or any Grade 5 event requires immediate notification by the site to the study coordinator and co-PIs. The co-PIs will convene within 24 hours by teleconference and decide whether the event necessitates a pause in further enrollment. If the team cannot convene to review the event within 24 hours, the medical officer will make the final decision.

Reporting requirements for the IRB/IEC will be followed as appropriate.

## Study Discontinuation

The trial may be discontinued at any time by the protocol team, sponsor, funding agency, Malawi regulatory authorities, or institutional review board/ethics committee. Please refer to Section 5.1 on Data Analysis, specifically the section on Analytical Methodology for Interim Analyses for more detail on the decision to discontinue the trial for safety.

# STATISTICAL DESIGN AND ANALYSIS

## DATA ANALYSIS

## Overview and General Design

In brief, we plan to conduct a facility-based, double-blinded, individually randomized, non-inferiority trial of 3 days (intervention) versus 5 days (control) of oral amoxicillin DT for chest-indrawing pneumonia.

The study will include 2,000 HIV-1 seronegative children aged 2 to 59 months presenting with chest-indrawing pneumonia at KCH or BDH in Lilongwe, Malawi. The treatment groups for the chest-indrawing pneumonia study will include treatments with oral amoxicillin DT in two divided doses based on age bands (500 mg/day for children 2 months up to 12 months, 1000 mg/day for children 12 months up to 3 years, and 1,500 mg/day for children 3 years up to 5 years of age) for 3 days (+ 2 days placebo) versus 5 days. Treatment will be block randomized (with concealed block size) within each age group to achieve a 1:1 ratio of intervention and control.

## Randomization and Blinding Procedures

Randomization will be perform as 1:1 based on permuted blocks of concealed size within strata defined by age groups (2 up to 12 months, 12 up to 36 months, 36 up to 59 months). Three randomization lists (one for each age group) will be provided by the statisticians to the study pharmacy. The study pharmacists and the Protocol Statisticians will be the only individuals who have access to the treatment assignment for each study patient. Patients, their caregivers and all other study personnel will remain blinded during the course of the study until the primary results manuscript is finalized.

The Sponsor does not anticipate any type of emergency situation where unblinding a child’s randomization allocation would be necessary. If an unanticipated need to unblind a child’s randomization allocation for reasons of participant safety arises during the course of the study, the site investigators, sponsor, and IRB/IEC will be notified and the instance will be documented.

## Objectives and Endpoints

In this study, the primary null hypothesis will be that the primary outcome of treatment failure at 6 days in those who received 3 days of amoxicillin DT (and 2 days placebo DT) is inferior (non-inferior in the alternative hypothesis) to those who received 5 days of amoxicillin DT for the treatment of chest-indrawing pneumonia in children aged 2–59 months. Eligible children will be randomly assigned to receive oral amoxicillin DT twice daily for 3 days (and 2 days placebo) in the intervention group and for 5 days in the control group. The children will be evaluated on day 4, after 3 days of treatment, to assess response to treatment and on day 6, after 5 days of treatment, to assess treatment failure; by this time point, they would have received all their treatment and we would expect them to either be cured or to have failed treatment. They will also be evaluated on days 2 and 14 to assess response to treatment. If a child becomes ill again after the chest-indrawing initially returned to normal, they will be encouraged to return between days 6 and 14 to assess for relapse.

The primary outcome will be treatment failure before or on day 6 for intervention and control groups. Secondary outcomes include: (a) clinical relapse between days 6 and 14 if treatment failure was not present before or on day 6, (b) combined rates of clinical relapse and treatment failures before or on day 14, (c) AEs and SAEs, (d) prevalence of malaria among children with cough and/or difficulty in breathing AND fever, (e) differential treatment response among children with wheeze at screening, (f) differential treatment response among children with oxygen saturation <93% by pulse oximetry at baseline, (g) differential treatment response among children with MUAC-defined moderate malnutrition at baseline, (h) differential treatment response among children with very fast-breathing for age at baseline, and (i) differential treatment response by age. Refer to Section 3 on Hypothesis, Objectives, and Endpoints for full description of all objectives.

- *Treatment failure* will be defined as development of any of the following criteria during the specified time periods:
  - Any time before or on day 6: WHO IMCI danger signs, severe respiratory distress (e.g., grunting, nasal flaring, head nodding, or severe chest-indrawing), oxygen saturation by pulse oximetry < 90%, missing 3 or more doses due to vomiting, change in antibiotics prescribed by a study clinician, or death.
  - At or after initial hospitalization discharge assessment (between 42 and 60 hours post-enrollment): documented axillary temperature > 38 ºC with chest-indrawing.
  - At day 6 outcome assessment: persistence of chest-indrawing, or axillary temperature > 38 ºC.
  - For the purposes of this protocol, children who do not fail on assessment at day 6 will be considered clinically cured. Loss to follow-up or withdrawal from the study at any time after enrollment and before the day 14 follow-up visit will be considered missing outcome data for that respective time point.
- *Clinical relapse* will be defined as recurrence of signs of chest-indrawing pneumonia, severe respiratory distress (e.g., grunting, nasal flaring, head nodding, or severe chest- indrawing) or severe disease after day 6 among those who did not have treatment failure at or by day 6.

Recruitment and follow-up is expected to continue until the maximum sample size is achieved. For the chest-indrawing pneumonia study a total of 2,000 children (1,000 per treatment group) will be enrolled. We are assuming a treatment failure rate of 8% in the 5-day control group and a relative 1.5 non-inferiority margin. Figure 3 shows the maximum true failure rate in the 3-day treatment group in reference to various possible failure rates in the 5-day treatment group and a 1.5 relative non-inferiority margin (50% relative increase in failure rate) that would result in a 95% confidence interval at maximum enrollment which excludes the non-inferiority margin. The figure also indicates the power the study has to show non-inferiority if the failure rates are exactly the same in the two treatment groups for various possible failure rates. Of note, the estimated power is adjusted for a 5% loss to follow-up rate in each of the arms. This is considered conservative with respect to the multiple imputation procedure that will be used to account for missing outcome values.

**Figure 3.** Chest-indrawing pneumonia failure rates

Note:

- Blue, solid circles, blue number below: Potential failure rates for the 5-day treatment group.
- Blue, solid circles, blue number above: Power to detect the alternative of exactly equal failure rates in both treatment groups
- Green, hollow squares: Non-inferiority margins (depending on assumed failure rate in the 5-day treatment group.
- Hollow, red circles: Maximum true failure rate for the 3-day treatment group observed at maximum enrollment of 2,000 children (1,000 children in each control and treatment groups and assuming 5% loss to follow-up in each of the groups resulting in 1,900 children with complete outcome data for the primary analysis) for which the 95% confidence interval excludes the non-inferiority margin. For example, for a failure rate of 8% in the 5-day treatment group (assumed for the chest-indrawing pneumonia), the true failure rate in the 3-day treatment group can be 9.7% (1.7% above the control group) and can still rule out with high confidence a failure rate of 12% in the 3-day treatment group.

## Analytical Methodology for Interim Analyses

For the chest-indrawing cohort, we plan two interim analyses after about one-third and two-thirds of children have been enrolled. We assume a one-sided test with an alpha=0.025, sample size=1900 (assuming 5% loss to follow-up in each of the arms), a Pocock design for early inferiority and O’Brien-Fleming for early non-inferiority stopping boundaries and a 1.5% relative difference failure rate between treatment (3-day) and control (5-day amoxicillin). The DSMB will consider recommending to stop the study prior to maximum enrollment if they determine early non-inferiority, early inferiority, or safety concerns. Details of the sequential monitoring plan will be finalized in collaboration with the DSMB and provided in the Statistical Analysis Plan (SAP).

## Analytical Methodology for Final Analyses

We expect the project to produce evidence for chest-indrawing pneumonia that supports the effectiveness of 3 days of amoxicillin DT treatment to be non-inferior to 5 days of amoxicillin DT treatment.

We anticipate that some children may not return for their scheduled follow-up visits if no specific measures are taken to encourage more complete follow-up. In addition to appointment reminders and counseling caregivers on the importance of completing follow-up, study staff will provide incentives and transportation costs to minimize missing outcome data. For those children who do not return for their scheduled follow-up visits, study staff will conduct home visits the next day to assess the outcome. Even with these measures in place, we have estimated the loss to follow-up to be 5%. Children lost to follow-up cannot be classified as improved or treatment failures at the missed visit. We will use multiple imputations for any missing outcome data and perform sensitivity analyses to assess how our results might change if the imputation assumptions are changed in a reasonable way, informing the robustness of the primary analysis result. We will perform complete case analysis as one form of sensitivity analysis. The imputations will be performed separately for each treatment group and cohort using multiple (20) hotdeck imputations and adherence information as well as child’s age, gender, literacy status of the caregiver, and number of children in the household.

For the primary outcome, we will estimate the difference in failure rate between the 3-day and 5-day amoxicillin DT treatment groups (after adjustment for age, 2 up to 12 months, 12 up to 36 months and 36 up to 59 months) and calculate a 95% CI. The 3-day treatment will be considered non-inferior to the 5-day treatment group if the upper level of the 95% confidence interval excludes a relative increase in failure rate of 50% (factor of 1.5).

To address potential misclassification of eligibility and outcome, we plan to include sub-studies to validate eligibility and outcome in 10% of randomly selected children. For more details on potential misclassification and associated analyses, refer to the SAP.

## RESULT PRESENTATION

The results of this research will be primarily presented through at least one published manuscript with detailed description of the background, methods, results, and conclusion. The specific format and details of this manuscript will be in accordance with the requirements of the publishing journal, but is expected to include tables describing the baseline characteristics of study participants and the differences between randomization arms for each study endpoint.

## DISSEMINATION OF RESULTS

The results of this study will be published collaboratively by investigators at Save the Children Federation, Save the Children International, the University of Washington, the University of Malawi, and the Ministry of Health in peer-reviewed journals. Study findings will be presented to the Malawi MOH Senior Management and hospital staff at the study site. Co-investigators plan on attending at least one international conference to disseminate the findings of the study.

# ETHICAL CONSIDERATIONS AND CONSENT

## Principles for Clinical Research

This clinical trial will be conducted in compliance with the protocol, International Conference on Harmonization Good Clinical Practice E6 (ICH-GCP), and all applicable regulatory requirements and Institutional Review Boards/Independent Ethics Committee (IRB/IEC) reviews. All study staff will be trained and certified in the protection of human subjects.

## Institutional Review Boards (IRBs) and Independent Ethics Committees (IECs)

The IRB and IEC of record for this clinical trial are the Western Institutional Review Board (WIRB) and the University of Malawi College of Medicine Research and Ethics Committee (COMREC). A copy of the protocol, proposed informed consent forms, other written participant information, and any proposed advertising material will be submitted to both WIRB and COMREC for written approval. The investigators must submit and, where necessary, obtain approval from the IRB/IEC for all subsequent protocol amendments and changes to the informed consent document. The investigators will notify the IRB/IEC of serious adverse events according to the IRB/IEC requirements. The Sponsor, CRO, and study operations partner (SC) are responsible for assuring that this protocol and the associated informed consent documents and study-related documents are approved by WIRB and COMREC prior to implementation of the protocol. Any amendments to the protocol, informed consents, or other study-related documents must be approved by the IRB/IEC prior to implementation. The study will be conducted in full compliance with the protocol. Any deviations from or violations of the protocol will be documented and submitted to the IRB/IEC by investigators as required. The protocol will not be amended without prior written approval by the PI and Sponsor.

## Informed Consent

In obtaining and documenting informed consent, the site investigators and their designees will comply with applicable local and domestic regulatory requirements and will adhere to GCP and to the ethical principles that have their origin in the Declaration of Helsinki. This clinical trial will have an informed consent form (ICF) for screening and an ICF for enrollment developed for local use that are in accordance with all applicable regulations. Both an English and Chichewa version of the ICFs will be reviewed and approved by the IRB/IEC of record before use with participants. The consent forms will include the purpose of the study, the investigational products to be administered, a description of the procedures to be followed and the risks and benefits of participation. The informed consent process will give individuals all of the relevant information they need to decide whether to participate, or to continue participation, in this study. Potential research participants’ caregivers will be permitted to ask questions and to exchange information freely with the study team. If the caregiver providing consent is illiterate, an independent witness will be present to verify to the caregiver that all the information read aloud is contained in the ICF. In this instance, both the caregiver and witness will sign the ICF.

Before a child begins participation in the study, it is the site investigators’ responsibility to ensure that informed consent is obtained from a LAR after adequate explanation of the aims, methods, and potential risks and benefits of the study. The study staff obtaining consent will also sign and date the ICF. A signed and dated copy of the consent form will be given to the participant’s caregiver and this will be documented in the child’s record.

## Risks to Participants

- - Randomization arms

This is a randomized trial that is investigating the appropriate duration of treatment with oral amoxicillin DT for pneumonia. It is possible that 3 days and 5 days of amoxicillin treatment are not equivalent for managing chest-indrawing pneumonia and that participants receiving the shorter course of antibiotics could suffer a higher treatment failure rate, with an increased risk of adverse events, re-hospitalization or death. Those children in the 5-day amoxicillin arm may have received antibiotics that were unnecessary, increasing their exposure to antibiotics and the potential risks of medication side effects.

- - Coercion

Caregivers may feel coerced to enroll in the study in order to receive care for their child within a research setting, which may be perceived as of a higher quality than the standard of care.

- - Specimen Collection

The study involves blood specimen sampling at screening. Phlebotomy can cause pain and bruising at or around the blood draw site.

- - Medical Management

Participation in the study has the potential to compromise care for hospitalized children, if study procedures are prioritized above urgent clinical care for acute infections.

## Protection against Risks

- - Randomization arms

In order to minimize the risk of adverse events, treatment failure, hospitalization, and death, eligibility criteria for this study have been carefully selected and a robust safety monitoring scheme is in place. The children with pneumonia most at risk of treatment failure and/or death will be excluded from this study, including those with WHO IMCI danger signs and HIV infection or exposure. Safety monitoring for this study includes frequent clinical examination at study visits for the first four days, outcome assessment and a clinician on call via an emergency hotline for the first fourteen days, treatment and tracking of all AEs and SAEs, and an external DSMB for regular review of cumulative safety and study conduct data. Refer to Section 4.10 of this protocol for more detail on the study’s Safety Assessments and Reporting procedures.

- - Coercion

In order to minimize the risk of coercion, study staff will not be recruiting participants directly. Instead, OPD clinicians will inform caregivers about the study and refer only those who are interested. During the informed consent process, study staff will emphasize that the child will receive medical care whether enrolled in the study or not.

- - Specimen Collection

In order to minimizing the risks associated with phlebotomy, all study staff who will be collecting specimens from children in the study will be trained in the appropriate procedures and supervised accordingly.

- - Medical Management

In order to minimize the possibility that participation in this trial will interfere with the medical management of children with pneumonia at KCH, study staff will have the primary responsibility for the clinical management of hospitalized children. Hospitalized children (e.g., during the first two days) will be treated and managed by study staff in accordance with standard procedures. Study staff will be informed about any decisions regarding treatment failure and changing antibiotic regimens made by KCH staff. Please refer to Section 4.6 Study Procedures for further description of Management of Study Participants During Hospitalization.

## Benefits to Participants

Direct benefits to children in this trial include increased clinical supervision and care during the study period as compared to alternatives not in a study setting. Frequent follow-up visits are not included as standard of care, so participating children will benefit from monitoring for two weeks from the pneumonia episode. This supervision will make it more likely that a case of treatment failure is identified and managed accordingly as compared to in a non-study setting. Additionally, participants’ caregivers will have access to a 24/7 hotline, answered by trained staff, which is not a part of standard of care.

If this trial demonstrates non-inferiority of 3 days of oral amoxicillin, the results have great potential to inform and support national and international guidelines for duration of treatment for childhood pneumonia.

## Participant Confidentiality

The site investigators must ensure that the child’s confidentiality is maintained. Personal identifiers will not be included in any study reports. All study records will be kept confidential to the extent provided by national and local laws.

All study procedures will be conducted to protect participant privacy and confidentiality to the fullest extent possible. The study site will establish a standard operating procedure (SOP) for confidentiality protection that includes both clinic and home visits and reflects the input of study staff and community representatives to identify potential confidentiality issues and strategies to address them.

## Participant Reimbursement

Travel reimbursement will be provided to caregivers to compensate them for the cost of transport for study visits. Reimbursement will be approximately the local currency equivalent of US$5.00 for each scheduled hospital-based study visit, payable at the end of the visit. Reimbursement for interim study visits will be approximately the local currency equivalent of US$2.50. The reimbursement amount may be modified during the course of the study to reflect potential changes in participant costs. The study consent form will list the minimum amount to be paid in the local currency. Participants’ caregivers will not receive reimbursement for visits that occur while the child is hospitalized to avoid disruptions in the hospital wards with other non-study patients.

Participants’ caregivers will receive a phone card with airtime worth MK 100 on the carrier of their choice (either AirTel or TNM) to cover any phone calls the caregiver may need to make to study staff during the course of the study.

Study participants’ caregivers will not be responsible for paying for study-related drugs, tests, or examinations.

## Storage of Specimens

Specimens collected during the course of this research will not be stored. Any leftover samples not consumed during study-related diagnostic tests will be destroyed.

# POSSIBLE CONSTRAINTS

Anticipated implementation challenges to the successful outcome of the study include:

1. Ensuring quality and consistency of implementation at the trial site. We plan to provide standardized training, supervision, and oversight to ensure quality and harmonized trial procedures. A CRO will be contracted to provide additional oversight and monitoring of the site, as needed.
2. Enrolling 2,000 children with chest-indrawing pneumonia within the anticipated timeline. Recognizing that chest-indrawing pneumonia is less common than fast-breathing pneumonia, we anticipate that enrollment for the chest-indrawing pneumonia trial might take longer to complete. To address this concern, we have decided to conduct the trial at a very high-volume health facility to maximize enrollment.
3. Following up all children. Recognizing that some children may not come back for the follow-up visits, we plan to include and train study staff to locate children who miss their follow-up appointments and conduct these visits in the home. We will also ensure that study staff take the time to educate caregivers on the importance of adhering to the treatment regimen and follow-up.

# REQUIREMENTS AND TRAINING

See Appendix VII for a description of study requirements for study activities, including training for study personnel and KCH staff.

# REFERENCES

1. Graham K. (2014) Study of community case management of pneumonia in Zambia: factors which determine rational use of antibiotics. *Malaria Consortium*.
2. Grant GB, C. H. (2009). Recommendations for treatment of childhood non-severe pneumonia. *Lancet Infect Dis, 9*, 185-196.
3. Kabra SK, L. R. (2010). Antibiotics for community-acquired pneumonia in children. *Cochrane Database of Systematic Reviews* (3).
4. Addo-Yobo E, C. N. (2004). Oral amoxicillin versus injectable penicillin for severe pneumonia in children aged 3 to 59 months: a randomised multicentre equivalency study. *Lancet, 364*, 1141-1148.
5. Atkinson M, L. M. (2007). A multicentre randomised controlled equivalence trial comparing oral amoxicillin and intravenous benzyl penicillin for community acquired pneumonia in children PIVOT Trial. *Thorax, 62*, 1102-1106.
6. Hazir T, F. L. (2008). Ambulatory short-course high-dose oral amoxicillin for treatment of severe pneumonia in children: a randomised equivalency trial. *Lancet, 371*, 49-56.
7. Bari A, S. S. (2011). Community case management of severe pneumonia with oral amoxicillin in children aged 2–59 months in Haripur district, Pakistan: a cluster randomised trial. *Lancet, 378*(9805), 1796-1803.
8. World Health Organization. (2014). *Revised Guidelines for Pneumonia Case Management at Health Facilities: Scientific Evidence.* Geneva: World Health Organization.
9. United Nations Development Programme (UNDP). (2014). *Country Profiles, Malawi.* UNDP.
10. F, D. (2009). Epidemiology of Malaria in Malawi. In B. C. Geubbels E, *The Epidemiology of Malawi.* University of Malawi: Division of Community Health, College of Medicine.
11. United Nations AIDS Program (UNAIDS). (2014). *Country Progress Reports, Malawi.* UNAIDS.

# Protocol Appendices

# Protocol Appendix I: Schedule of study visits and evaluations

|  | SCREEN-ING | ENROLL-MENT  Day 1 | Day 2 | Day 4 | Day 6 | Day 14 | Interim visit(s) |
| --- | --- | --- | --- | --- | --- | --- | --- |
| Informed Consent | ✓ | ✓ |  |  |  |  |  |
| Comprehension Checklist |  | ✓ |  |  |  |  |  |
| Participant ID | ✓ | ✓ | ✓ | ✓ | ✓ | ✓ | ✓ |
| Eligibility Assessment | ✓ | ✓ |  |  |  |  |  |
| Demographics | ✓ | ✓ |  |  |  |  |  |
| Locator Information | ✓ | ✓ | ✓ | ✓ | ✓ | ✓ | ✓ |
| Randomization |  | ✓ |  |  |  |  |  |
| Reimbursement |  | ✓ |  | ✓ | ✓ | ✓ | ✓ |
| Schedule Next Visit | ✓ | ✓ | ✓ | ✓ | ✓ |  |  |
| Medical History | ✓ | ✓ | ✓ | ✓ | ✓ | ✓ | ✓ |
| Targeted Physical Exam | ✓ | ✓ | ✓ | ✓ | ✓ | ✓ | ✓ |

# Protocol Appendix II: Sample collection and laboratory evaluations

|  | Specimen | Screen-ing | Enroll-ment  (Day 1) | Day 2 | Day 4 | Day 6 | Day 14 | LABORA-TORY |
| --- | --- | --- | --- | --- | --- | --- | --- | --- |
| ITIP2 Diagnostic | HIV test | ✓ |  |  |  |  |  | Study site |
|  | Anemia test | ✓ |  |  |  |  |  |  |
|  | Malaria test | ✓ |  |  |  |  |  |  |

# Protocol Appendix III: Study requirements and training

Additional study requirements not already described in the protocol are summarized below.

- **Personnel**

The study team on-the-ground will consist of full-time employees in the following capacities:

- - Study coordinator: clinician who will oversee daily study operations and monitor the safety of participants
  - Study medical officers/clinical officers: clinicians whose daily work will involve screening potential study participants, enrolling children into the study, performing clinical assessments during observation periods, clinically managing hospitalized study participants, and conducting the safety monitoring and reporting for adverse events in the study patients.
  - Pharmacists: receive, account for, prepare, and distribute study product; train study staff on study product administration procedures.
  - Data manager: maintain study databases with quality control and quality assurance procedures; prepare regular reports of ongoing study activities and data.
  - Data officers/assistants: scan data collected on paper forms for entry into electronic database(s), maintain copies of study documents as needed, prepare study documents and ensure all in correct format for study participant files, perform quality control checks in the study participants’ CRF`s.
  - Study nurses: perform screening, enrollment, follow-up and interim study visit procedures, conduct informed consent process, conduct all home visits and study retention efforts.
  - HTC counselors: perform HIV test pre-counseling, testing and post-test counseling per study protocol and Malawi national guidelines.
  - Fieldworkers: perform home follow up visits.

In addition to the full-time staff, the project will use the expertise of additional Save the Children (SC) personnel to assist with meeting study goals. A portion of SC staff’s time will be for operational and grant management services, which will provide a range of support activities to the project such as accounting, human resources management, information technology, administration and audit services.

Government staff at the study site hospital will also be engaged with this study. KCH service providers will be responsible for recruiting of study participants at KCH or BDH and managing participants’ care while in the hospital.

- **Training**

All study staff will be trained in the Protection of Human Subjects prior to any interactions with study participants. Additionally, before the study starts, all study staff will attend an extensive 5-day study-specific training to review all study procedures, including the study protocol, SOPs, data collection tools, informed consent process, reporting requirements, and safety monitoring. Refresher trainings on the identification of pneumonia will be scheduled at least once per year and will include updates from the study monitor reports. Trainings will be conducted by a Sponsor representative, representative of the study CRO, or other qualified clinician, as appropriate for the training material.

Government staff at KCH and BDH will be sensitized to this study and will receive at least one day of training on the identification of pneumonia and study-specific procedures and documentation prior to the study start. Refresher trainings will be held periodically, at least once every year.

- **Supplies**

Supplies for this study include the following:

- - Laptops for study staff
  - Printer
  - Photocopier
  - Respiratory rate counters
  - Portable pulse oximeter
  - Scale
  - Height board
  - Malaria RDT kits
  - Office furniture
  - Partitions/privacy screens for the study clinic
  - Communication equipment such as cellphone accessories, airtime, and internet sticks
  - Standard office supplies, including binders, paper, pens
- **Transportation**

The study will obtain multiple motorcycles for use by the study retention team to conduct home visits after a participant misses a scheduled study visit. Study participants will be expected to provide their own transportation to study visits at KCH, but will receive a travel reimbursement.

- **Space**

The study clinic for out-patient screening and enrollment will be located in the OPD of KCH or BDH. The study clinic for follow-up and interim visits will be located in the Pediatric Department of KCH. The hospital has provided the study with a private room for study visits and other study-related activities. Additional office space for data management and the study coordinator will be provided at a separate location in Lilongwe.

# Protocol Appendix IV: Study sensitization/recruitment script

**Instructions:** This script is to be used by Kamuzu Central Hospital and Bwaila District Hospital staff in the Paediatric Outpatient Department after the initial triage and intake of a presenting child. This content should be presented to caregivers of children who are between 2 and 59 months of age and have cough or difficult breathing.

**Script:** “There are two ongoing research studies for children with pneumonia and your child may be eligible to participate in one of them. The studies are investigating different treatment regimens for childhood pneumonia, seeing if less antibiotic use is as effective for curing pneumonia. If you are interested in learning more about these studies, I can let the study staff know that it is okay to contact you. If you aren’t interested in the studies, that is fine and no one from the study will contact you about them. Your decision to participate in a study will not affect the medical care that your child receives in the hospital. Are you interested in learning more about the studies?”

**Prompts:** If families ask other questions about the study, including procedures, risks, or benefits, they should be referred to study staff.

**Statistical Analysis Plan**

“**Innovative Treatments in Pneumonia (ITIP) 2**:

Double-blind randomized controlled clinical trial of 3 days versus 5 days amoxicillin dispersible tablets for chest-indrawing childhood pneumonia among children 2-59 months of age presenting to Kamuzu Central Hospital in Lilongwe, Malawi”

Co-Principal Investigators: Amy Ginsburg (PATH) & Salim Sadruddin (Save the Children)

Study Biostatistician: Susanne May (University of Washington)

Statistical Analysis Plan prepared by: Rob Schmicker (University of Washington)

Version 1.0, August 24, 2015

Version 1.1, January 5, 2017

1. **INTRODUCTION**

Pneumonia is responsible for more than one in five child deaths around the globe. Each year, approximately 1.1 million children die before their fifth birthdays due to pneumonia, more than the number of under-five deaths that result from human immunodeficiency virus (HIV), tuberculosis, and malaria combined. In addition to preventing pneumonia, there is a critical need to provide greater access to appropriate and effective treatment. Studies in Asia have evaluated the effectiveness of 3 days of oral amoxicillin for the treatment of fast-breathing pneumonia; however, further evidence is needed to determine if 3 days of oral amoxicillin is also effective for the treatment of chest-indrawing pneumonia. Finally, given the paucity of data from Africa, African-based research is necessary to establish optimal treatment regimens for childhood pneumonia in the region.

With the expressed support of the Malawi Ministry of Health (MOH) and in collaboration with external experts from the University of Washington (UW), PATH and Save the Children Federation, Inc. (SC) will work closely with an investigator at the College of Medicine (COM) at the University of Malawi to build evidence regarding appropriate duration of treatment with amoxicillin dispersible tablets (DT) for chest-indrawing childhood pneumonia in malaria-endemic settings in Africa. An expanded evidence base will contribute to future iterations of integrated community case management guidelines, which in turn will test innovative approaches to childhood pneumonia treatment.

The proposed approach involves conducting a double-blinded, randomized, non-inferiority trial with the objective to assess the effectiveness of shorter duration amoxicillin DT treatment of chest-indrawing childhood pneumonia in a malaria-endemic region of Malawi.

1. **ANALYSIS OBJECTIVES**
   1. Primary Objective
      1. To determine whether 3 days of treatment with oral amoxicillin DT in HIV-negative children 2 to 59 months of age with chest-indrawing pneumonia is as effective as 5 days of treatment.
   2. Secondary Objectives
      1. To determine whether the intervention arm has equivalent rates of treatment relapse as the control arm among those without treatment failure before or on day 6.
      2. To determine whether the intervention arm has equivalent rates of combined treatment failure and relapse before or on day 14 as the control arm.
      3. To determine whether the intervention arm has equivalent rates of adverse events (AEs) and serious adverse events (SAEs) as the control arm.
      4. To investigate whether there may be a differential treatment response in children who test positive for malaria at baseline. This information will be useful to plan further childhood pneumonia and malaria integrated interventions in similar settings.
      5. To determine whether there is a differential treatment response in enrolled children with wheeze during screening (identified prior to any bronchodilator administration).
      6. To determine whether there is a differential treatment response in enrolled children with an oxygen saturation level <93% by pulse oximetry at baseline.
      7. To determine whether there is a differential treatment response in enrolled children with mid-upper arm circumference (MUAC)-defined moderate malnutrition at baseline (11.5-13.5cm).
      8. To determine whether there is a differential treatment response in enrolled children with very fast-breathing for age at baseline (>70 breaths per minute for 2–11 months, >60 breaths per minute for 12–59 months).
      9. To determine whether there is a differential treatment response by age.
2. **DESIGN**

This project involves a double-blinded, randomized, non-inferiority trial in children 2-59 months of age from a malaria-endemic setting in Malawi comparing the effectiveness of 3-day to 5-day amoxicillin DT treatment for chest-indrawing, community-acquired pneumonia (ITIP2).

We plan to evaluate treatment with 3 days versus 5 days of oral amoxicillin DT in two divided doses based on age bands (500 mg/day for children 2 months to 12 months, 1000 mg/day for children 12 months to 3 years, and 1,500 mg/day for children 3 years to 5 years of age) among 2,000 children presenting with chest-indrawing pneumonia in a malaria-endemic region of Malawi. Children in the 3-day arm will also receive 2 days of placebo following 3 days of active drug to complete a 5-day course to maintain the blind as to their randomization allocation.

1. **DATA SOURCE**

All study data will be collected by the clinical study staff using designated source documents or electronic case report forms (CRFs). Study data will be entered directly into the electronic CRFs during a study visit. Paper-based versions of all CRFs will also be available for use in the event that electronic data capture is not possible from a technical standpoint. In that case, data will be entered after the fact into the electronic database as promptly as is feasible.

Clinical research data will be maintained through a combination of secure electronic data management system and physical files with restricted access. Triclinium Clinical Trial Project Management (Pty) Ltd. will serve as the contract research organization (CRO) and will be responsible for primary data management activities during the trial. Among other responsibilities, the data management activities include data entry and validation, data coding and cleaning, and database quality control.

Data related to study endpoints will be extracted from the electronic databases for statistical analysis. Two distinct study databases will be available to extract for statistical purposes: the primary study database with study visit data and a safety database with SAE assessments. Statisticians will not have access to any database with participating children’s personally identifiable information. The two study databases containing study endpoint data will identify children only by study identification numbers.

Statisticians will have access to a data download from the web based database at a minimum on a weekly basis.

1. **ANALYSIS SETS/POPULATIONS/SUBGROUPS**

Children age 2-59 months who present to Kamuzu Central Hospital or Bwaila District Hospital in Lilongwe, Malawi with chest-indrawing will be screened for participation in the study. Study staff will perform screening procedures to determine eligibility. They will assign a participant screening number and will collect demographic information, medical history and eligibility criteria data. To be eligible for participation in the study, children must meet the following inclusion/exclusion criteria:

5.1 Inclusion Criteria

- - Male or female, 2 to 59 months of age.
  - History of cough <14 days or difficult breathing with chest-indrawing.
  - Ability and willingness of child’s caregiver to provide informed consent and to be available for follow-up for the planned duration of the study, including accepting a home visit if he/she fails to return to KCH for a scheduled study follow-up visit.

5.2 Exclusion Criteria

- If chest-indrawing observed at screening resolves after bronchodilator challenge, among those with wheeze at screening.
- Severe respiratory distress (e.g., grunting, nasal flaring, head nodding, or severe chest-indrawing).
- Presence of WHO IMCI danger signs including: lethargy or unconsciousness, convulsions, vomiting everything, or inability to drink or breastfeed.
- Hypoxia (SaO_2_ < 90% on room air, as assessed by a Lifebox pulse oximeter).
- Stridor when calm.
- HIV-1 seropositivity or HIV-1 exposure, assessed as follows:
  - An HIV-positive result upon rapid antibody test will exclude any child from this study.
  - If a child is less than 12 months or age with a positive rapid test result, the child will be referred to receive additional confirmatory HIV testing (e.g., dried blood spot filter paper test) and follow-up from KCH staff, as per standard of care. Even if the confirmatory HIV testing subsequently shows that child is HIV-negative, he or she will remain excluded from the study.
  - If a child is less than 24 months of age and has an HIV-negative result upon rapid antibody test, the child’s biological mother’s HIV status will need to be assessed. If the mother is HIV-positive, the child will be excluded. If the mother has a documented HIV-negative test result from within the past 6 weeks, the child will be included. If the mother does not have documentation of an HIV-negative test result, she will be tested via rapid antibody testing to determine the child’s eligibility for this study.
  - If a child is over 24 months of age, an HIV-negative rapid antibody test is required for inclusion in the study.
  - Note: If a child has documentation of an HIV-negative test result from within the past 6 weeks, that test result will be used for the child’s eligibility assessment according to the algorithm described above.
- Severe acute malnutrition (i.e., weight for height/length < -3 SD, mid-upper arm circumference <115 mm, or edema).
- Possible tuberculosis (coughing for more than 14 days).
- Severe anemia, classified by WHO pocketbook guidelines (i.e., severe palmar pallor or hemoglobin <8.0 g/dL)
- Severe malaria, classified by WHO pocketbook guidelines (e.g., positive mRDT with any danger sign, stiff neck, abnormal bleeding, clinical jaundice, or hemoglobinuria)
- Known allergy to penicillin or amoxicillin.
- Receipt of an antibiotic treatment in the 48 hours prior to the study based on caregiver’s self-report and/or documentation in child’s medical record.
- Hospitalized within 14 days prior to the study.
- Living outside Lilongwe urban area, the study catchment area.
- Any medical or psychosocial condition or circumstance that, in the opinion of the investigators, would interfere with the conduct of the study or for which study participation might jeopardize the child’s health.
- Any non-pneumonia acute medical illness which requires antibiotic treatment per local standard of care.
- Participation in a clinical study of another investigational product within 12 weeks prior to randomization or planning to begin participation during this study.
- Prior participation in an Innovative Treatments in Pneumonia study during a previous pneumonia diagnosis.

Once a child is enrolled in the study, the staff will collect further data such as baseline characteristics, vital signs, vaccination history and additional socio-economic information. Caretakers will bring enrolled children in for follow-up visits on days 2, 4, 6 and 14. Upon follow-up, study staff will collect information on medical history since the previous visit, study product adherence and results from the physical exam.

Statisticians will have access to all appropriate study visit data on both enrolled patients and screened patients who were not enrolled.

*5.3 Subgroups*

Though listed in the protocol as secondary endpoints, we will examine the primary endpoint of treatment failure by day 6 in three specific subgroups:

1. Proportion of children with treatment failure among those testing positive for malaria by rapid diagnostic testing (mRDT) at baseline or outcome assessment (overall).
2. Proportion of children failing treatment among those with wheeze at baseline during screening (prior to administration of bronchodilators).
3. Proportion of children failing treatment among those children 2-12 months, 13-35 months, and 36-59 months of age at baseline.
4. Proportion of enrolled children failing treatment among those with oxygen saturation <93% by pulse oximetry at baseline.
5. Proportion of children failing treatment among those with MUAC-defined moderate malnutrition (11.5-13.5cm).
6. Proportion of children failing treatment among those with very fast breathing for age (>70 breaths per minute for 2–11 months, >60 breaths per minute for 12–59 months)

The same treatment failure definition as above will be used for these subgroups analysis. Subgroup classification will be obtained from the screening data.

Additional subgroups not defined in either the approved protocol or statistical analysis plan will be considered post-hoc. Statisticians will keep a list of post-hoc analysis requested by investigators during the study duration.

1. **ENDPOINTS AND COVARIATES**

*6.1 Primary endpoint*

The primary endpoint will be the proportion of children failing treatment, defined as the development of any of the following before or on day 6:

- - - - WHO Integrated Management of Childhood Illness (IMCI) danger signs
      - Severe respiratory distress (e.g., grunting, nasal flaring, head nodding, or severe chest-indrawing).
      - Oxygen saturation < 90% by pulse oximetry
      - Vomiting within 30 minutes of 3 or more scheduled (i.e., not repeat) dose administrations of study product
      - Change in antibiotics prescribed by a study clinician (e.g., switch to a second-line antibiotic or prescription for onset of a co-infection)
      - Death

At or after initial hospitalization discharge assessment (between 42 and 60 hours post-enrollment):

- - - - Documented axillary temperature > 38 ºC with chest-indrawing

At day 6 outcome assessment:

- - - - Documented axillary temperature > 38 ºC
      - Chest-indrawing

The primary endpoint will be calculated from day 2, 4, 6 follow-up forms. The presence of any of the above criteria at any point prior to day 6 will constitute treatment failure. In addition to the overall treatment failure rate by day 6, we will also examine the treatment failure rates at 2 and 4 for both treatment arms.

Covariates for primary analysis: In addition to the treatment variable, the primary analysis will include (be adjusted for) the following covariates: age (2 months up to 12 months, 12 months up to 3 years, 3 years up to 5 years) and site (Bwaila District Hospital and Kamuzu Central Hospital). Of note, these are the same variables that are used for stratifying randomization. In addition, we will adjust for gender, as gender has been shown to be related to pneumonia mortality^[1]^.

*6.2 Secondary endpoints*

The trial has the following several secondary endpoints

1. Proportion of children with clinical relapse between treatment failure assessment and day 14 follow-up visit among all children without treatment failure before or on day 6.
2. Proportion of children with either treatment failure or clinical relapse before or on day 14 (among all randomized children).
3. Proportion of children with AEs and SAEs.
4. Proportion of children with treatment failure among those testing positive for malaria by rapid diagnostic testing (mRDT) at baseline (overall).
5. Proportion of enrolled children failing treatment among those with wheeze during screening (identified prior to administration of bronchodilators).
6. Proportion of enrolled children failing treatment among those with oxygen saturation <93% by pulse oximetry at baseline.
7. Proportion of children failing treatment among those with MUAC-defined moderate malnutrition (11.5-13.5cm).
8. Proportion of children failing treatment among those with very fast-breathing for age (>70 breaths per minute for 2–11 months, >60 breaths per minute for 12–59 months).
9. Proportion of children failing treatment by age at baseline.
10. **STATISTICAL METHODOLOGY**

*7.1 Design*

The study will include 2,000 children aged 2 to 59 months. Children will be randomized to receive oral amoxicillin for 5 days (control) or 3 days + 2 days placebo (treatment). Treatment will be block randomized (with concealed block size) to ensure a 1:1 ratio of intervention and control. Randomization will be stratified by age group (2 up to 12 months, 12 up to 3 years, 3 years up to 5 years) and site (Bwaila District Hospital and Kamuzu Central Hospital).

*7.2 Interim analysis* We planned two interim analyses after about one-third and two-thirds of children have been enrolled. The seqDesign^[2]^ software was used to determine the appropriate non-inferiority and futility stopping boundaries at each analysis. We assumed a one-sided test with an alpha=0.025, sample size = 1900 (assuming 5% loss to follow-up in each of the arms), a Pocock design for early inferiority and O’Brien-Fleming for early non-inferiority stopping boundaries and a 1.5 relative non-inferiority boundary.

The maximum true failure rates at both the interim analyses and final analysis for the 3-day group for which the 95% CI excludes the non-inferiority margin for various 5-day treatment group failure rates are provided in Table 1 below.

Table 1. Maximum true failure rates for the 3-day treatment arm beyond which stopping for early non-inferiority will be considered

| 5-day failure rate | Non-inferiority bound | Max 3-day failure rate, 1^st^ interim analysis | Max 3-day failure rate, 2^nd^ interim analysis | Max 3-day failure rate, final analysis |
| --- | --- | --- | --- | --- |
| 7.0% | 10.5% | 4.0% | 7.2% | 8.3% |
| 8.0% | 12.0% | 5.0% | 8.5% | 9.7% |
| 9.0% | 13.5% | 6.0% | 9.8% | 11.0% |
| 10.0% | 15.0% | 7.1% | 11.1% | 12.4% |
| 11.0% | 16.5% | 8.2% | 12.4% | 13.7% |
| 12.0% | 18.0% | 9.4% | 13.7% | 15.1% |

The minimum true failure rates for the 3-day treatment group for which futility is considered for various 5-day treatment group failure rates are provided below.

Table 2. Minimum true failure rates for the 3-day treatment arm where futility will be considered

| 5-day failure rate | Min 3-day failure rate, 1^st^ interim analysis | Min 3-day failure rate, 2^nd^ interim analysis | Min 3-day failure rate, final analysis |
| --- | --- | --- | --- |
| 7.0% | 10.2% | 8.9% | 8.3% |
| 8.0% | 11.7% | 10.3% | 9.7% |
| 9.0% | 13.2% | 11.7% | 11.0% |
| 10.0% | 14.6% | 13.1% | 12.4% |
| 11.0% | 16.1% | 14.5% | 13.7% |
| 12.0% | 17.6% | 15.9% | 15.1% |

Figure 1 shows the maximum true failure rate in the 3-day treatment group in reference to various possible failure rates in the 5-day treatment group and a 1.5 relative non-inferiority margin (50% relative increase in failure rate) that would result in a 95% confidence interval at maximum enrollment which excludes the non-inferiority margin. The figure also indicates the power the study has to show non-inferiority if the failure rates are exactly the same in the two treatment groups for various possible failure rates. Of note, the estimated power is adjusted for a 5% loss to follow-up rate in each of the arms. This is considered conservative with respect to the multiple imputation procedure that will be used to account for missing outcome values.

**Figure 1.** Chest-indrawing pneumonia failure rates

Note:

- Blue, solid circles, blue number below: Potential failure rates for the 5-day treatment group.
- Blue, solid circles, blue number above: Power to detect the alternative of exactly equal failure rates in both treatment groups
- Green, hollow squares: Non-inferiority margins (depending on assumed failure rate in the 5-day treatment group.

Hollow, red circles: Maximum true failure rate for the 3-day treatment group observed at maximum enrollment of 2,000 children (1,000 children in each control and treatment groups and assuming 5% loss to follow-up in each of the groups resulting in 1,900 children with complete outcome data for the primary analysis) for which the 95% confidence interval excludes the non-inferiority margin. For example, for a failure rate of 8% in the 5-day treatment group (assumed for the chest-indrawing pneumonia), the true failure rate in the 3-day treatment group can be 9.7% (1.7% above the control group) and can still rule out with high confidence a failure rate of 12% in the 3-day treatment group.

The DSMB will consider recommending to stop the study prior to maximum enrollment if they determine early non-inferiority, early inferiority, or safety concerns.

Early non-inferiority will be considered if the 3-day failure rate is lower than the rates provided in Table 1 for a given 5-day treatment failure rate. For instance, if the 5-day treatment rate at the first interim analysis is 7%, then early non-inferiority will be considered if the 3-day failure rate is less than 4.0%.

Early futility will be considered if the 3-day failure rate is higher than the rates provided in Table 2 for a given 5-day treatment failure rate. For instance, if the 5-day treatment rate at the interim analysis is 7%, then early futility will be considered at the first interim analysis if the 3-day failure rate is higher than 10.2%.

- 1. *Final analysis*

For the primary outcome, we will estimate the failure rate difference and 95% CI between the 3-day and 5-day amoxicillin DT treatment groups after adjustment for age, 2 up to12 months, 12 months up to 3 years, 3 years up to 5 years, site (Bwaila District Hospital and Kamuzu Central Hospital) and gender (precision variable^[1]^). Since estimates are expressed in rate differences we will run a linear regression model with treatment arm as predictor (3-day vs. 5-day), failure rate (yes vs. no) as outcome and age, site and gender as covariates using robust standard errors and calculate a 95% CI. We will use a 1.5 relative non-inferiority margin. If the failure rate in the 5-day arm is 7%, the non-inferiority margin will be 10.5% (=7%*1.5). With a 5% failure rate the non-inferiority margin will be 7.5% (=5%*1.5).

To account for anticipated loss to follow-up or study withdrawal we will use multiple imputations for any missing outcome data. We will then perform sensitivity analyses to assess how the results might change with varying imputation assumptions. More information is provided in section 8 below.

1. **HANDLING OF MISSING DATA AND OTHER DATA CONVENTIONS**

We have estimated that loss to follow-up or study withdrawal will be 5%. Children lost to follow-up cannot be classified as improved or treatment failures at the missed visit. We will use multiple imputations for any missing outcome data. The imputations will be performed separately for each treatment group and cohort using multiple (20) hotdeck imputations and other information as described below. Multiple imputation estimates will be combined using Rubin’s approach^[3]^.

Specifically, for each episode that is missing outcome data, we will create a subset of episodes that match exactly on treatment arm and gender while matching on age category, highest educational level achieved by the mother (none, primary, secondary, tertiary, while combining these categories for categories with less than 5% of participants, if needed, unknown education level will be multiply imputed, independent from outcome imputation) and adherence information (all combinations of categories for

a) days 1-3: no missing doses, 1-3 missing doses, 4-6 missing doses and

b) days 4-5: no missing doses, 1-2 missing doses, 3-4 missing doses,

unknown dosing information will be conservatively imputed as “not taken” for the 3 day arm and similar to patients where dosing is available for the 5-day arm). We will then randomly select one episode from the corresponding subset with complete information and use its values to replace the entire episode with missing outcome data.

We will repeat this process for each episode missing outcome data and calculate failure rates for both treatment arms using the imputed outcome. We will repeat this 20 times and determine the mean failure rates for each treatment arm. The difference between the mean failure rates will be calculated. Noninferiority bounds as listed in 7.2 will be consulted.

1. **SENSITIVITY ANALYSIS**

We will perform sensitivity analyses to assess how our results might change if the imputation assumptions are changed in a reasonable way, informing the robustness of the primary analysis result. We will perform complete case analysis as one form of sensitivity analysis.

1. **PROGRAMMING PLANS**

All data cleaning and programming will be done in R^[4]^.

*10.1 DSMB Tables*

Data tables will be exported from R to Excel in the form of raw data. Raw data files will then be linked to a formatted Excel file and turned into PDF. Draft DSMB Open Session tables will be sent to appropriate clinical and statistical staff prior to the DSMB meeting and will use dummy randomization for the treatment arms. After finalizing content of DSMB tables, real randomization will then be used for the DSMB meeting.

*10.2 Manuscript tables*

Data tables will be exported from R to Excel in the form of raw data. Raw data files will then be linked to a formatted Excel file. Appendix A displays tables that will be considered for inclusion in the primary manuscript.

APPENDIX A – Tables

Table 1: Patient and Clinical Characteristics at Enrollment

|  | Arm A  (n=X) | Arm B  (n=X) | Overall  (n=X) |
| --- | --- | --- | --- |
| Age |  |  |  |
| Median (IQR) | xx (xx) | xx (xx) | xx (xx) |
| 2-12 months, n (%) | xx (xx%) | xx (xx%) | xx (xx%) |
| 13-36 months, n (%) | xx (xx%) | xx (xx%) | xx (xx%) |
| 37-59 months, n (%) | xx (xx%) | xx (xx%) | xx (xx%) |
| Sex |  |  |  |
| Male, n (%) | xx (xx%) | xx (xx%) | xx (xx%) |
| Female, n (%) | xx (xx%) | xx (xx%) | xx (xx%) |
| Weight (kg) |  |  |  |
| Median (IQR) | xx (xx) | xx (xx) | xx (xx) |
| Height/length (cm) |  |  |  |
| Median (IQR) | xx (xx) | xx (xx) | xx (xx) |
| Weight-Height Z-Score |  |  |  |
| Median (IQR) | xx (xx) | xx (xx) | xx (xx) |
| Vital Signs, median (IQR) |  |  |  |
| Axillary Temperature (°C) | xx (xx) | xx (xx) | xx (xx) |
| Oxygen Saturation (%) | xx (xx) | xx (xx) | xx (xx) |
| Respiratory Rate (breaths/min) | xx (xx) | xx (xx) | xx (xx) |
| Pulse Rate (beats/min) | xx (xx) | xx (xx) | xx (xx) |
| Caregiver Assessment |  |  |  |
| Diarrhea | xx (xx%) | xx (xx%) | xx (xx%) |
| Fever | xx (xx%) | xx (xx%) | xx (xx%) |
| Cough | xx (xx%) | xx (xx%) | xx (xx%) |
| Fast/difficult breathing | xx (xx%) | xx (xx%) | xx (xx%) |
| Chest-indrawing | xx (xx%) | xx (xx%) | xx (xx%) |
| Nasal blockage | xx (xx%) | xx (xx%) | xx (xx%) |
| Runny nose | xx (xx%) | xx (xx%) | xx (xx%) |
| Poor feeding | xx (xx%) | xx (xx%) | xx (xx%) |
| Feeling cold to touch | xx (xx%) | xx (xx%) | xx (xx%) |
| Vomiting | xx (xx%) | xx (xx%) | xx (xx%) |
| Lethargy | xx (xx%) | xx (xx%) | xx (xx%) |

Table 2: Treatment Failure Prior to Day 6

|  | Arm A  (n=X) | Arm B  (n=X) | Overall  (n=X) |
| --- | --- | --- | --- |
| Treatment Failure On or Before Day 6 | xx (xx%) | xx (xx%) | xx (xx%) |
| Reason for Treatment Failure |  |  |  |
| WHO Danger Signs | xx (xx%) | xx (xx%) | xx (xx%) |
| Hypoxia (SaO_2_ < 90%) | xx (xx%) | xx (xx%) | xx (xx%) |
| Axillary Temperature ≥ 38 in absence of  diagnosed co-infection w/ fever symptoms | xx (xx%) | xx (xx%) | xx (xx%) |
| Change in antibiotics | xx (xx%) | xx (xx%) | xx (xx%) |
| Prolonged hospitalization or re-admission  due to pneumonia | xx (xx%) | xx (xx%) | xx (xx%) |
| Chest-indrawing | xx (xx%) | xx (xx%) | xx (xx%) |
| Death | xx (xx%) | xx (xx%) | xx (xx%) |

Table 3: Treatment Failure Prior to Day 6 for Pre-Specified Subgroups

|  | Arm A  (n=X) | Arm B  (n=X) | Overall  (n=X) |
| --- | --- | --- | --- |
| Malaria Positive Test, n (%) | xx (xx%) | xx (xx%) | xx (xx%) |
| Malaria Negative Test, n (%) | xx (xx%) | xx (xx%) | xx (xx%) |
|  |  |  |  |
| Wheeze at baseline, n (%) | xx (xx%) | xx (xx%) | xx (xx%) |
| No wheeze at baseline, n (%) | xx (xx%) | xx (xx%) | xx (xx%) |
|  |  |  |  |
| Age 2-12 months, n (%) | xx (xx%) | xx (xx%) | xx (xx%) |
| Age 13-36 months, n (%) | xx (xx%) | xx (xx%) | xx (xx%) |
| Age 37-59 months, n (%) | xx (xx%) | xx (xx%) | xx (xx%) |

Table 4: Clinical Relapse on Day 14 by Treatment Arm

|  | Arm A  (n=X) | Arm B  (n=X) | Overall  (n=X) |
| --- | --- | --- | --- |
| Clinically Cured on Day 6 | xx | xx | xx |
| Clinical Relapse on Day 14, n (%) | xx (xx%) | xx (xx%) | xx (xx%) |
| Clinical Relapse Criteria or Danger Signs |  |  |  |
| Convulsions | xx (xx%) | xx (xx%) | xx (xx%) |
| Lethargic or unconscious | xx (xx%) | xx (xx%) | xx (xx%) |
| Unable to feed or drink |  |  |  |
| Vomits everything | xx (xx%) | xx (xx%) | xx (xx%) |
| Stridor when calm | xx (xx%) | xx (xx%) | xx (xx%) |
| Hypoxia (SaO_2_ < 90%) | xx (xx%) | xx (xx%) | xx (xx%) |
| Axillary Temperature ≥38 in absence of  diagnosed co-infection with fever symptons |  |  |  |
| Change in antibiotics |  |  |  |
| Prolonged hospitalization or re-admission  due to pneumonia |  |  |  |

References

1. Marzia Lazzerini, Nadine Seward, Norman Lufesi, Rosina Banda, Sophie Sinyeka, Gibson Masache, Bejoy Nambiar, Charles Makwenda, Anthony Costello, Eric D McCollum, Tim Colbourn, 2016, Mortality and its risk factors in Malawian children admitted to hospital with clinical pneumonia, 2001–12: a retrospective observational study, Lancet Global Health, 4, e57-68.
2. Scott Emerson, John Kittelson and Dan Gillen (2014). RCTdesign: Design, monitoring, and analysis of sequential clinical trials.. R package version 1.1.
3. Rubin DB. Multiple Imputation for Nonresponse in Surveys. Hoboken, NJ: Wiley Inc; 1987
4. R Core Team (2013). R: A language and environment for statistical computing. R Foundation for Statistical Computing, Vienna, Austria. URL http://www.R-project.org/.
